# Efficiency of spatio-temporal vaccination regimes in wildlife populations under different viral constraints

**DOI:** 10.1186/1297-9716-43-37

**Published:** 2012-04-24

**Authors:** Martin Lange, Stephanie Kramer-Schadt, Hans-Hermann Thulke

**Affiliations:** 1 Helmholtz Centre for Environmental Research Leipzig - UFZ, Dept. of Ecological Modelling, Leipzig, Germany; 2 Leibniz Institute for Zoo and Wildlife Research, Alfred-Kowalke-Str. 17, Berlin D-10315, Germany

## Abstract

Classical Swine Fever (CSF) is considered an endemic disease in European wild boar populations. In view of the high economic impact of the introduction of the virus into domestic pig units, huge efforts are invested in the preventive control of CSF in wild boar populations. Recent European Community guidelines favour oral mass vaccination against CSF in wild boar populations. The guidelines are explicit on the temporal structure of the vaccination protocol, but little is known about the efficacy of different spatial application schemes, or how they relate to outbreak dynamics.

We use a spatially explicit, individual-based wild boar model that represents the ecology of the hosts and the epidemiology of CSF, both on a regional scale and on the level of individual course of infection. We simulate adaptive spatial vaccination schemes accounting for the acute spread of an outbreak while using the temporal vaccination protocol proposed in the Community guidelines.

Vaccination was found to be beneficial in a wide range of scenarios. We show that the short-term proactive component of a vaccination strategy is not only as decisive as short-term continuity, but also that it can outcompete alternative practices while being practically feasible. Furthermore, we show that under certain virus-host conditions vaccination might actually contribute to disease persistence in local populations.

## Introduction

Disease outbreaks in wildlife populations often have huge economic consequences for the livestock industry [1,2] or pose an enormous risk to public health [3-6]. Managing diseases in wildlife populations is therefore of paramount importance [7]. Oral mass treatment is one method of choice in wildlife disease control [8-12]. Mass vaccination, for example, has largely succeeded in eradicating rabies in Central Europe [13-15]. Although the success of large-scale control efforts has been demonstrated repeatedly in the field [16-20], other studies show that wildlife diseases persisted for decades despite huge control efforts [21,22].

In contingency strategy planning, research has very much focused on the level of treatment coverage required for herd-immunity or disease fade-out [23-29] or on temporal aspects such as the timing of campaigns in relation to seasonal reproduction in wildlife [20,29-34]. Particularly in the design of wildlife mass treatment programmes spatial aspects may play an important role in relation to species' dispersal abilities, spatial heterogeneity and spatio-temporal disease spread [35-37]. Integrating existing knowledge of the host's ecology and behaviour into application strategies is crucial for optimising the control of wildlife diseases [38].

Classical Swine Fever (CSF) in wild boar populations is a prominent example. In recent years, the virus circulated in wild boar in several European countries. Having entered livestock, it resulted in severe economic losses both for individual farmers and for national economies [1,39,40]. Wild boar are considered a virus reservoir and the main source of infection for domestic pig farms through direct contact and swill feeding [40]. Enormous effort has been invested in oral vaccination campaigns, hygiene and hunting measures during outbreaks in several countries. However, the effects of these measures on disease dynamics are not always fully understood [29]. Hunting, particularly of juvenile boars, was considered an effective measure against CSF by reducing the densities of susceptible hosts, but later turned out to be a factor potentially encouraging virus persistence through compensatory breeding and boar dispersal [18,29,41-43].

Current research on oral mass vaccination using hand-distributed baits at feeding sites still shows contradicting results [20,29,44]. In some cases oral mass vaccination was shown to eradicate the infection [18,20,45], while in other cases mass vaccination did not lead to disease extinction [21,22]. This difference is attributed to the highly dynamic host population over space and time [46,47], the involvement of virus strains with different virulence [48] and the variability of the disease outcome between infected individuals [49,50]. As a consequence, there is still a debate about the most reasonable control aims of mass vaccination in wild boar, the usefulness of marker vaccines to monitor control in the field, or the most plausible spatio-temporal design for vaccination protocols [29,51]. In this context, Rossi et al. [20] were able to demonstrate for a forest area in France that preventive vaccination, i.e. vaccinating the unaffected population around an outbreak, is more effective than treating infected areas only.

The purpose of this study is the systematic investigation of spatially differentiated baiting regimes with regard to their efficacy in limiting the spread and survival of the infection in a wild boar population. The systematic comparison of vaccination schemes requires consideration of large spatial and temporal scales. Moreover, the complex interaction of host ecology and behaviour, infection dynamics, variable virulence and control effects limits potential of empirical experimentation. Process-based epidemiological modelling can capture biological variability and uncertainty with their delicate balance among potentially counteracting effects [41,52-54]. We use a validated ecological-epidemiological model and compare current spatial baiting strategies with alternative schemes. We show why current baiting schemes have suboptimal control characteristics and, how control could profit from orally applicable marker vaccines [41,51]. Moreover, we specify epidemiological conditions under which artificial immunisation through oral vaccination can even facilitate disease persistence.

## Materials and methods

### Model description

#### Overview

Our model is based on the approach by Kramer-Schadt et al. [53] and is implemented in FreePascal/Lazarus. The individual-based, spatially explicit ecological-epidemiological model was parameterised with field data. It is described according to the ODD protocol (overview, design, details [55,56]). Submodels are presented where essential for understanding the model output or where modified from the literature version. Complete submodel descriptions are given in the Additional file 1.

#### Purpose

The ecological-epidemiological model was used to investigate spatially dynamic bait distribution schemes of oral mass vaccination of wild boar populations against CSF with special emphasis on their efficacy in limiting the spread and survival of the infection.

#### State variables and scales

The model comprises two major components: a wild boar demography model considering seasonal reproduction, dispersal and mortality, and a CSFV model operating on the emerging boar population. Boar population density and structure are affected by the disease via lethality and litter size depression (see Additional file 1 Section "Reproduction").

The crucial model entity is the boar individual, characterised by age in weeks, resulting in the age classes piglet (< 8 months ± 6 weeks), yearling (< 2 years ± 6 weeks) and adult. Each host has a location, which also denotes its home range cell as well as the individual's family group. Further host state variables are demographic status (disperser or non-disperser), epidemiological status (susceptible, transiently infected or lethally infected with varying survival times, or immune by surviving the infection, vaccination or maternal antibodies) and sex.

The model landscape is represented by a grid of 4 km^2 ^cells, each of which encompass a boar family group's core home range [57]. Each grid cell is characterised by a breeding capacity, denoting habitat quality and thus the maximum possible number of female boars that can breed (i.e. population density regulation). Family groups are formed by females and new ones are established by seasonal subadult dispersal. Males although associated as individuals to cells do not disperse explicitly as females are known to be responsible for the spatial distribution and establishment of wild boar [47]. The foraging movement of a wild boar group extends beyond the core area and spatial overlap allowing for e.g. transmission of an infection between families.

#### Process overview and scheduling

The model proceeds in weekly time steps which represents the approximate CSF incubation time [58]. Processes of each time step are infection, wild boar group splitting, reproduction, death and ageing, executed in the given order.

In the first week of each year, mortality probabilities are assigned stochastically to represent annual fluctuations in boar living conditions. Then every boar is assigned as breeder or non-breeder, according to the carrying capacity of its home range cell.

### Design concepts

Boar population dynamics emerge from individual behaviour, defined by age-dependent seasonal reproduction and mortality probabilities and age- and density-dependent dispersal behaviour, all including stochasticity. The epidemic course emerges from within and between group virus transmission, boar dispersal and individual courses of infection. Stochastic lethality of infection and stochastic infectious periods are modelled explicitly, since variation in disease outcome between individuals was known as essential for virus endemicity in host populations [53].

### Details

#### Initialisation

The heterogeneous model landscape with an extent of 200 km × 50 km is initialised randomly with uniformly distributed integer breeding capacity values *C_ij _*∈ {0, 1,...,5}. The average breeding capacity is 2.5 females per cell, resulting in approximately 20 boars per cell or a host density of 5 boars per km^2^. Boar density reflects long-term average values of densely populated Central European boar habitats [42,59,60] reviewed in [61]. The landscape thus corresponds to connected boar habitat without barriers with a population size of about 50 000 individuals, corresponding European wild boar populations [18,62,63]. The rectangular shape of the landscape with a high longitudinal, compared to the transversal extent, was chosen to allow for the analysis of disease spread and its velocity, while keeping computational costs tractable.

One boar group is released to each habitat cell. Initial group size is three times the breeding capacity. Initial age distributions are taken from the results of a 100-year model run conducted by Kramer-Schadt et al. [53].

#### Submodels

In this section we describe the submodels required for the simulation experiments. More detailed submodel descriptions are given in the Additional file 1.

##### Transmission

**Figure 1:**
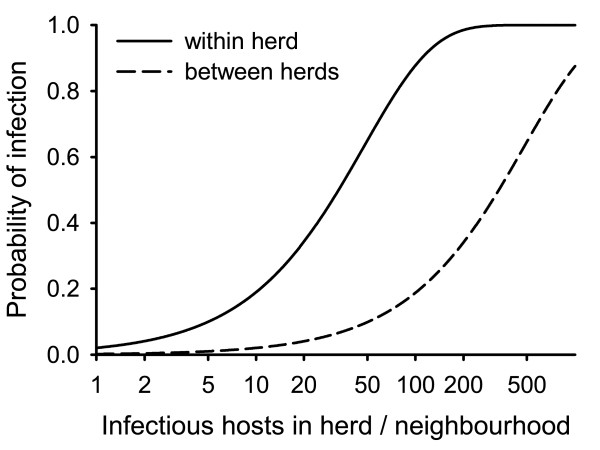
**Probability of infection, depending on the number of infectious hosts in the herd (solid, **$P_{\inf}^{\left(i\right)}=2.08\cdot 10^{-2}$**) and in neighbouring herds (dashed, **$P_{\inf}^{\left(e\right)}=2.08\cdot 10^{-3}$**)**.

Transmission is modelled stochastically. Parameters determine the probability of contracting the infection from an infectious group mate $P_{\inf}^{\left(i\right)}$ and the probability of contracting the infection from an infectious animal in a neighbouring group $P_{\inf}^{\left(e\right)}$ (3 × 3 neighbourhood) during one week. For each susceptible animal the probability of becoming infected accumulates over all infectious animals within the group and in the neighbourhood:

$$\Pi_{i}=1-{\left(1-P_{\inf}^{\left(i\right)}\right)}^{\lambda_{i}}\cdot {\left(1-P_{\inf}^{\left(e\right)}\right)}^{\sum_{j}\lambda_{j}}$$

where *λ_i _*is the number of infected individuals in the herd and *λ_j _*is the number of infected individuals in herds of the 8 neighbouring cells *j*. The model iterates over all individuals and stochastically sets each susceptible individual to infected if a uniformly distributed random number *r *drawn from *U*(0, 1) is smaller than ∏*_i _*of its home cell (Figure 1).

**Figure 2:**
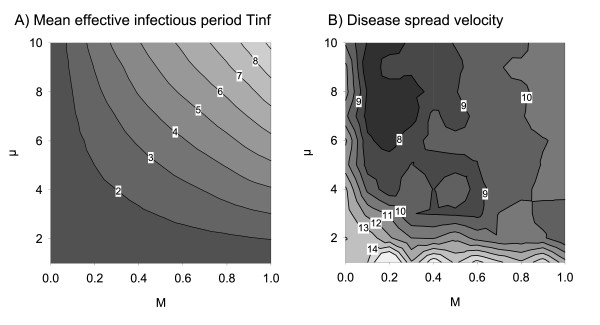
**Isoclines of (A) mean effective infectious period *T*_inf _[weeks], corresponding to parameter combinations *M *× *μ *and averaged over all infected animals**. (B) Disease spread velocity [km/quarter].

The transmission parameter was reversely fitted [64,65] to the estimated average disease spread velocity of approx. 8 km per quarter [20] using linear regression (v = -1.5 + 454.5 * $P_{\inf}^{\left(i\right)}$, R^2 ^= 0.21). The resulting parameter values were assigned constant as $P_{\inf}^{\left(i\right)}=2.08\cdot 10^{-2}$ within and $P_{\inf}^{\left(e\right)}=2.08\cdot 10^{-3}$ between groups. Resulting spread velocities over the entire parameter space are shown in Figure 2b. The resulting probability of infection, depending on the number of infectious hosts in the herd and in the neighbourhood is shown in Figure 1.

Infection might be regularly translocated within the host population during the dispersal of subadult females.

##### Disease course

**Figure 3:**
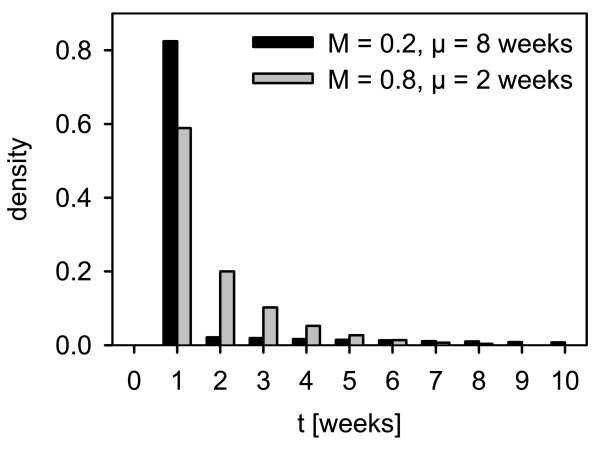
**Realisation of infectious periods over transiently and lethally infected hosts for *M *= 0**.2, *μ *= 8 weeks (black) and *M *= 0.8, *μ *= 2 weeks (grey).

The disease course submodel is described by two parameters: first, individual case mortality (*M*) and second the mean infectious period of lethally infected hosts (*μ*). Both are used to decide stochastically on the individual disease course of an infected host i.e. whether it recovers after a transient infection or when it dies. In detail, the parameter individual case mortality (*M*) is defined as the Bernoulli probability that decides whether the infection of an individual host is lethal (with probability *M*) or runs transient (1-*M*). *M *applies unchanged for yearlings *M*^(y) ^= M, is decreased for adults to *M*^(a) ^= *M*^2 ^and increased for piglets to $M^{\left(p\right)}=\sqrt{M}$ to represent age-dependent disease outcomes [66]. Transiently infected boars are infectious for one week, then protected before they turn to the immune state three weeks later [58,67]. The infectious period (in weeks) of lethally infected hosts is drawn from an exponential distribution with mean *μ*. Figure 3 shows two examples of the resulting distribution of infectious periods summarizing lethally and transiently infected hosts. Lethally infected hosts remain infectious until death.

##### Vaccination

**Figure 4:**
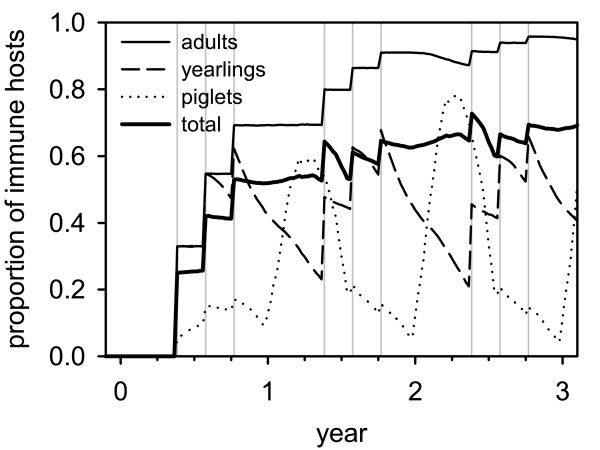
**Time series of proportion of immune hosts due to oral mass vaccination (total and for age classes)**. Vertical grey lines denote dates of baiting campaigns.

Temporal schedule of vaccination campaigns over each year was equivalent for all simulations and followed existing protocols [29]: baiting campaigns are possible in fixed calendar weeks 20, 30 and 40 (end of May, early August, mid-October). Vaccination starts after virus release with the first possible campaign according to the temporal schedule and ends with the end of the simulation. Spatial application schemes were experimentally altered and are described in the section "Simulation experiments". Bait uptake rates in terms of the proportion of hosts that receive at least one bait per campaign were assigned as 33% for adults and yearlings, and 5% for piglets. Piglet uptake rates were set low to represent difficulties in the vaccination of juveniles, particularly those younger than 4 - 5 months [12,29]. Bait uptake is evaluated independent of the animals' epidemiologic or vaccination status. The resulting devolution of the proportion of immune hosts over time in a susceptible population is shown in Figure 4 and mimics respective field observations [18]. Immunity is life-long, and no booster effect is implemented in the model. Oral vaccination in wild boar is performed recently with C-strain filled baits. This modified live vaccine was repeatedly demonstrated to provide sterile immunity in all animals after eating a bait [41,45]. A marker vaccine which has yet been used experimentally for oral vaccination in the field has equivalent protective characteristics [51].

## Parameters, simulation experiments, analysis

### Parameters

For parameter values and sources of submodels described in this document, see Table 1. For the complete list of parameters, see Additional file 1.

**Table 1:** Parameters of the submodels.

Symbol	Description	Value	Source/details
*T_trans_*	Infectious period of transiently infected hosts	1 week	[58,67]

$P_{\inf}^{\left(i\right)}$	Infection probability within/between herds	2.08·10^-2^	Reversely fitted to the estimated disease spread velocity of approx. 8 km per quarter [20]
$P_{\inf}^{\left(e\right)}$		2.08·10^-3^	

*M*	Case mortality (subadults, see section "Disease course")	(0.0, 0.1, ..., 1.0)	

*μ*	Expectation value of the exponential distribution of life expectancy of lethally infected hosts	(1, 2, ..., 10) weeks	

*u*^(*a*)^	Bait uptake rates adults/yearlings/piglets	0.33	Piglet uptake rates low to represent difficulties in vaccination of juveniles, younger than 4-5 months [12,60].
*u*^(*y*)^		0.33	
*u*^(*p*)^		0.05	

For the complete list of parameters see Additional file 1.

### Independent variables

There are two independent variables in our analysis. Lethality of infection is defined by individual case mortality parameter *M*, and life-expectancy of lethally infected hosts is specified by the parameter *μ*. All simulation experiments were performed for *M *∈ {0.0, 0.1,...,1.0} and *μ *∈ {1, 2,...,10} to cover a wide range, no matter whether the extremes are biologically meaningful. The range covers lethality from 0 - 100% and mean infectious periods after lethal infection as long as 10 weeks. With that, we recognize the reported heterogeneity in these two parameters as measured in the field [68-70].

The individual disease courses were scaled up to the whole population to measure the effective mean infectious period *T*_inf_. The resulting isoclines of *T*_inf _are comprised in Figure 2a.

### Simulation experiments

**Figure 5:**
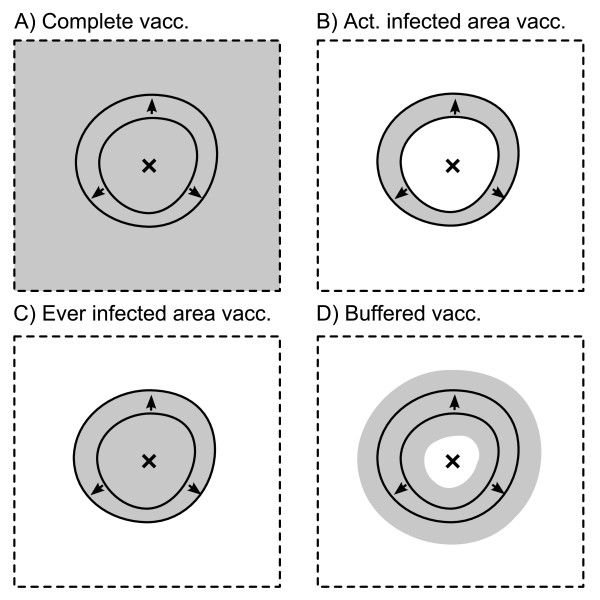
**Schematic diagram of spatial vaccination strategies with infected area (solid outlines), baiting zone (grey fill), virus release point (cross) and disease spread direction (arrows)**. The circular pattern of infected areas is idealized from more distorted model outcomes.

Four spatial vaccination schemes (Figure 5), and a non-vaccination reference were applied. The vaccination schemes were motivated by different level of accuracy in following the actually infected area:

0. "No vaccination": reference with no baiting at all.

1. "Complete vaccination": baiting is applied to the entire landscape (Figure 5a).

2. "Actually infected area vaccination": baiting is applied on all habitat cells that are infected in the week of the recent campaign (Figure 5b).

3. "Ever infected area vaccination": baiting is applied on all habitat cells that have been infected in the given model run (or recent outbreak, Figure 5c). The strategy is comparable to recent baiting strategies of successive vaccination zone extending with disease spread.

4. "Buffered vaccination": baiting is applied on all habitat cells that are infected in the week of the campaign and a buffer of 32 km around them (Figure 5d). The buffer radius of 32 km is motivated by the saturation of the proportion of immune hosts after three campaigns, i.e. one year (Figure 4) and the spreading velocity of the epidemic wave of 8 km per quarter, i.e. 32 km per year.

For each vaccination scheme and each *M *× *μ *combination 120 model runs were conducted to achieve a minimum precision of ± 9% with 95% confidence for proportions, resulting in 13 200 runs per scheme.

Simulations were performed for 20 years or until host or virus became extinct. In detail, the virus was released into the boar population in a random week of the sixth year by infection of one randomly selected boar individual and then simulations continued up to maximum further 14 years.

### Dependent variables

The simulation output focused on two dependent variables: (1) the extent of the outbreak as measured by the maximum distance from the release point, and (2) the risk of endemicity as measured by the probability of virus circulation after 10 years.

Maximum virus distance from the release point *D*_max _was recorded as a measure of disease spread. The average maximum distance from a randomly selected release point was about 155 km and is defined by the most distant corner of the landscape. In detail, for a landscape of 200 km × 50 km, average maximum edge distances are 150 km and 37.5 km, resulting in an average possible distance of $\sqrt{{(150\;\text{km)}}^{2}+{(37.5\;\text{km)}}^{2}}=154.6\;\text{km}$ if spread always covers the full landscape.

Virus persistence was measured in weeks since virus release. Individual runs were labelled endemic if the virus is present after 10 years and non-endemic for earlier virus fade-out. The proportion is then described by the dependent variable *P*_end _measuring probability of endemicity from 120 repetitions of a simulation scenario.

### Analysis

Data was analysed by applying contour plots of response variables using *M *and *μ *as X resp. Y axis. To identify the parameter scopes of the different effects of the schemes tested, differences to the reference scenario were calculated.

Analysis was performed using GNU R 2.9.2 (R Core Development Team); plots were created with SigmaPlot^® ^10.0 (Systat Software Inc.).

## Results

### Reference scenarios

**Figure 6:**
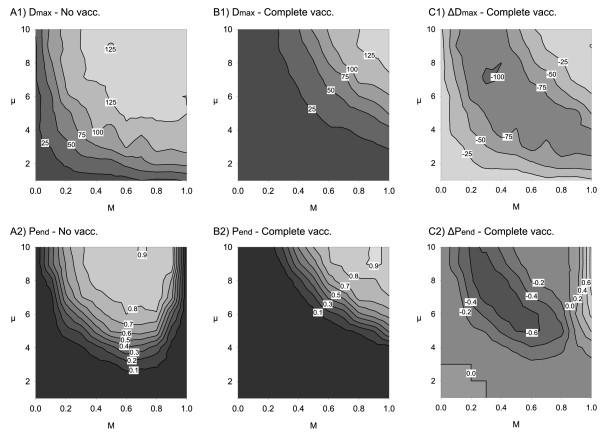
**(A) No vaccination, (B) complete vaccination, (C) pointwise difference between B and A**. (A1, B1, C1) Median of the maximum achieved distance of the virus from the release point *D*_max _without vaccination, with vaccination, and the amount of change between both. (A2, B2, C2) probability of virus endemicity *P*_end _without vaccination, with vaccination and the amount of change between both. All are plotted vs. case mortality *M *and mean infectious period of lethally infected hosts *μ*.

Before applying spatially structured vaccination effort, the model output was considered for the two reference scenarios: no vaccination (scenario 0) and complete area vaccination (scenario 1). The scenarios represent the extremes of possible vaccination effort. The model output was used to determine evaluation criteria for the two independent variables: *D*_max _and *P*_end_. Figure 6 shows the complete output data of all parameter combinations for non-vaccination (Figure 6a) and complete area vaccination scenario (Figure 6b), and the difference between the values of both scenarios, i.e. the effect of vaccination (Figure 6c).

Without vaccination the majority of parameter combinations revealed a high spreading potential, *D*_max_, of the infection (Figure 6a1). *D*_max _was limited only for very low case mortality or short survival times of lethally infected hosts (bottom or left in Figure 6a1). The negative values in Figure 6c1 indicate for which parameter combinations complete area vaccination shortened *D*_max_. Maximum reduction of *D*_max _by vaccination (Figure 6c1) was found for parameter combinations of *M *and *μ *in a bow shaped central area that overlays those between the isoclines for *T*_inf _= 2 from below and *T*_inf _= 6 above in Figure 2a. In the remaining parameter combinations, vaccination had a limited but negative, i.e. desired, impact on *D*_max_.

Turning to the probability of endemicity, Figure 6a2 revealed a cup-shaped central area where the infection became endemic after passing through the population. Here, the parameters comprise intermediate case mortality values *M *together with intermediate to long infectious periods of lethally infected hosts *μ*. For all other parameter combinations the probability of endemicity was nearly zero (Figure 6a2, right, bottom, left). This is reasonable as either maximum case mortalities *M *cause strong population thinning behind the epidemic front or minimum case mortality and very short infectious periods *μ *exclude bridging of local deficits of susceptible hosts due to the short average effective infectious period *T*_inf_. Complete area vaccination removed the cup-shape picture of likely endemicity (Figure 6b2). Vaccination decreased *P*_end _for *M *< 0.6 and intermediate to long *μ *(Figure 6c2). For the same range of *μ *but maximum case mortality *M*, vaccination increased *P*_end_. If infectious period of lethally infected hosts *μ *was shorter than 3 weeks *P*_end _already was zero and hence vaccination could not have any effect.

### Structured vaccination schemes

Next, the three alternative spatial vaccination schemes were considered with regard to the resulting changes in the model output measures *P*_end _and *D*_max_.

**Figure 7:**
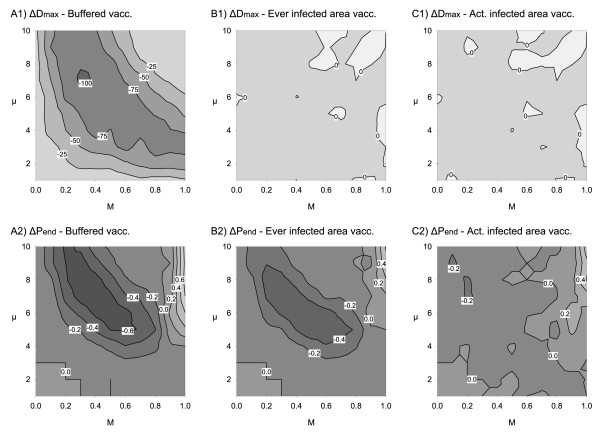
**Effect of strategies (A) buffered, (B) ever infected area and (C) actually infected area vaccination**. Top row: changes in maximum virus spread distance *D*_max_, bottom row: changes in probability of virus endemicity *P*_end_; plotted vs. case mortality *M *and mean infectious period of lethally infected hosts *μ*. Parameter combinations with negative difference reflect a positive impact of vaccination, i.e. reduced spreading distance or lowered probability of endemicity, while positive differences indicate a worse outcome of the vaccination alternative compared to the non-vaccination reference.

Buffered vaccination (scenario 4) had the strongest impact on both *D*_max _and *P*_end_. Noteworthy, the effect was almost identical to that of the complete area vaccination (cf. Figure 6c1 and 6c2 with Figure 7a1 and 7a2).

The impact of vaccinating the ever infected area (scenario 3; Figure 7b1 and 7b2) was found to be marginal in limiting spatial spread. However, the resulting changes in probability of endemicity roughly coincided with buffered vaccination but with an overall reduction in the effect strength (positive and negative change; Figure 7b2).

The impact of vaccinating the actually infected area (scenario 2; Figure 7c1 and 7c2) was found to be marginal both with regard to spatial spread and endemicity, only extreme case mortality *M *slightly increased the probability of endemicity (Figure 7c2, top right).

## Discussion

The presented simulation study provides understanding of how vaccination may impact the spread and maintenance of an infection in wild boar populations under different viral constraints [29]. The course of a potential outbreak was represented by an established model of Classical Swine Fever (CSF) in wild boar populations [53]. Therein, alternative spatial application schemes of oral mass vaccination were implemented. The comparative evaluation of alternatives was focused on two output quantities: (1) the extent of the outbreak was measured by the maximum distance from the release point, and (2) the risk of endemicity was measured by the probability of virus circulation after 10 years. The first measure is essential for planning the extent of restriction zones for the pig production sector according to existing disease legislation (EU Council Directive 80/217/EEC [71]). The second measure prescribes whether spatially restricted host populations can maintain the infection and, hence, translates into the necessity of disease control within an infected population [29,41,44,72-74]. Indeed, following the epidemic phase of an outbreak, either recurrent outbreaks have to occur inside parts of the area that already had been affected ("endemic phase" according to [29]), or the infection will fade out after spreading trough the population. The first defines endemicity as used in our analysis. The latter, although leading only to a transient infection of the host population, often also is referred to as "long" persistence because depending on the size of the connected and populated wild boar area, the epidemic phase of an outbreak might take a long time till fade out. For example, with a simulation area of 200 km × 50 km and spatial spread calibrated to 32 km per annum [20] an infection without potential to become endemic might still be present for 3-6 years depending on the place of introduction. Therefore, instead of referring to infections "persisting for a long time" in a population, we used "probability of endemicity" to describe possible maintenance of the infection after it had spread through the population.

Effectiveness of population vaccination is paralleled to the reduction in the number of secondary cases produced by e.g. an infected wild boar group [75,76]. The number depends on the probability of transmission and the average infectious period of infected individuals [77]. Under different viral constraints the latter might be highly variable between individual cases or different virus strains. For example, different experimental and field studies of CSFV exhibited a variety of outcome with regards to the lethality of individual infections (represented in the model by the parameter *M*) and the variability in the survival time of lethally infected hosts (from acute to rather chronic infections; represented by the parameter *μ *in the model) [29]. To cope with this uncertainty, the simulation results were produced by systematically sampling over the possible values of the two parameters that define individual disease courses. Simulations in the same wild boar population but assuming different viral constraints (Figure 6a2), subjected the full range from fade out (e.g. highly virulent strain CSFV/1.1/dp/CSF0382/XXXX/Koslov [78]) to endemicity (e.g. moderately virulent strain CSFV/2.3/wb/CSF1045/2009/Roesrath [78]) to the same vaccination schemes enabling most general comparison of effectiveness. Moreover, the results of the comparative evaluation were tested for their sensitivity to qualitative alteration of transmission probability (see Additional file 2) but were regained in full.

### Complete area vaccination and the maximum outcome

Compared to the simulations without vaccination (Figure 6a) the performance of vaccination was overviewed by the reduction of *P*_end _and *D*_max _for the different viral constraints (Figure 6c1 + 2). The results showed that maximum reduction of spatial spread of the infection was achieved for viral constraints that resulted in an effective average infectious period *T*_inf _between 2 and 6 weeks (Figure 2a). Thereof maximum reduction in the probability of endemicity was achieved only for moderate case mortalities (*M *< 60%) combined with sufficiently long infectious periods of lethally infected hosts (*μ *> 3 weeks). Interestingly recent outbreaks are characterised by moderate lethality of less than 50% and few acute courses i.e. few of the lethally infected animals dying within less than four weeks [41,44,58,62]. Thus, with the recent CSFV strains in wild boar (e.g. moderately virulent strain CSFV/2.3/wb/CSF1045/2009/Roesrath [78]) the treatment of the total wild boar population - although impractical - is expected to prevent spatial spread and the endemicity of the infection.

If high case mortality and long mean infectious periods of lethally infected animals were assumed, vaccination had no effect on the spatial spread but induced a prolongation of virus circulation. This is reasonable as without vaccination the decline of the population density behind the epidemic front due to high lethality already favours fade-out although spatial spread covered the whole area (Figure 6a1 + 2). Indeed, the initial average density of the simulated population was 5 hosts per km^2^, lethality of 80% left an approximate population density of 1 host per km^2 ^behind the epidemic front. Guberti et al. [26] estimated the threshold for CSFV spread as 1 host per km^2 ^making self-eradication a consistent outcome. Vaccination now prevented the collapse of the host population. As a consequence, the infection became endemic when the mean life expectancy of lethally infected hosts was set sufficiently long (i.e. *μ *above 4 weeks).

With CSFV such highly lethal infections are expected to coincide with rather short mean life expectancies of infected hosts (e.g. highly virulent strain CSFV/1.1/dp/CSF0382/XXXX/Koslov [78]). Therefore, such a negative impact of vaccination is less likely with CSFV control. More general, however, our results suggest that vaccination planning should be performed with caution if an outbreak is reported to kill the majority of infected animals: If the mean infectious period of the disease is very short, the outbreak is expected to be self-limiting in the population; if not, vaccination might even create endemicity.

### Alternative baiting strategies - feasible expectations

Astonishingly, the buffered vaccination approach backed up the efficacy of complete area vaccination with regard to both criteria "prevention of disease spread" (Figure 7a1 and Figure 6c1) and "eradication" (Figure 7a2 and Figure 6c2). Baiting one year ahead of disease spread sufficiently mimicked the large proactive component of complete area vaccination. The backward component of the buffered strategy shows parallels with recent control proposals [29] that recommend repeated vaccination for at least one year after the last case detection in a local area.

The key assets in the strategy are the temporal raise of population immunity and the spreading distance of CSFV during one year:

Considering the devolution of population immunity by number of campaigns in a susceptible population (Figure 4), the assumed bait uptake led to saturation of population immunity after about 3 campaigns (i.e. 1 year of regular vaccination schedule). This dynamics is comparable to the dynamics observed in areas where oral vaccination was practiced before the reporting of CSF infections [18].

In the simulation model, the known spreading velocity value of 8 km per annual quarter [20] was scaled to transmission between a sufficient number of wild boar groups. If, however, the spreading distance of 32 km per annum is less general, e.g. an infection spreads differently fast in other eco-regions, then the results of our simulations are robust and will be repeated if the width of the buffer is aligned to the alternatively reported distance value. The success of this strategy might be favoured by the regular boar habitat structure in the model. Whether alteration of spreading velocity in structured landscapes of boar habitat requires adjustment of buffer width needs further detailed examination [79-81].

Baiting of areas that were ever infected during the outbreak is comparable to recent baiting strategies of successive extension of vaccination zones in accordance with disease spread. The strategy has a strong backward component but no proactive component. Hence, it is reasonable that this strategy cannot decrease spatial disease spread. With continued, uncontrolled spread of the infection through the wild boar area the strategy successively converged to the complete area design along with an increasing proportion of the landscape affected. Virus eradication can thus be expected to take place in late-stage disease control, while annual baiting costs increase with ongoing disease presence. More relevant, however, is the lacking potential to prevent disease spread i.e. the growth of the infected area.

Baiting of actually infected habitat areas (Figure 7c) was revealed to be completely ineffective. This appears reasonable as both the proactive and the backward component of the complete area baiting are no longer realised. Although the approach is often taken to be most cost effective [82], this judgement usually ignores total effort, which accumulates quickly if no eradication can be achieved.

At first glance, these findings contradict vaccination successes reported from the field with ever infected area vaccination [18]. However, in this particular area the vaccination protocol changed during the control program [83] because the unavailable marker property of the applied vaccine limited the follow up investigation. Vaccination started with a wide buffer around the infected area (compare Figures 26a and 27a in von Rüden [83]), but later protocols foresee only the newly infected area to be added to the baiting area (ever infected area vaccination scenario) which did not actually happen. Following our findings, the positive effect of the "buffer" included in the initial campaigns already might have caused the observed success.

The concept of vaccinating the infected area or an additional buffer around it obviously depends on the monitoring of the infected area [60]. If monitoring relies on serological investigations (e.g. CSF), oral mass vaccination will hamper the applicability of the strategies if no marker vaccines are available for oral application which allow differentiation of vaccinated from infected animals. For vaccination against CSFV infections in wild boar efforts are on the way to substitute the recent vaccine by an orally applicable marker version with the same protective characteristics. The vaccine already was tested experimentally in the field and thus motivated the systematic evaluation of possible new spatial vaccination schemes that make use of the dynamic actual infected area (see, e.g. [51,84,85]).

## Competing interests

The authors declare that they have no competing interests.

## Authors' contributions

ML implemented the model and performed the simulation experiments and drafted the manuscript. SKS and HHT contributed to the modelling and experimental design of the study. All authors read and approved the final manuscript.

## Supplementary Material

Additional file 1 **ODD Model Documentation**. Click here for file

Additional file 2 **Sensitivity analysis**. Click here for file
