# Supplementary material for: Efficiency of spatio-temporal vaccination regimes in wildlife populations under different viral constraints
Source: Vet Res. 2012 Apr 24;43(1):37. doi: 10.1186/1297-9716-43-37 (PMC3384476; doi:10.1186/1297-9716-43-37)
Supplement: Additional file 1 — ODD Model Documentation. [file 1297-9716-43-37-S1.DOC]

**Supplementary material**

**Appendix 1: ODD Model Documentation**

# Overview

The CSF wild boar model is a compilation of a spatially explicit, stochastic, individual based demographic model for wild boars (*Sus scrofa*) and an infection and disease course model for the CSFV. The model is documented following the ODD protocol (Overview, Design, Details [1,2]).

## Purpose

The model aims to assess and optimise oral mass vaccination and other control measures against CSF in wild boar on population level.

## State variables and scales

The model comprises two major components, a wild boar demography model considering seasonal reproduction, dispersal and mortality, and a CSF virus model operating on the boar population. Boar population density and structure are inﬂuenced by the disease via virus-induced mortality and litter size depression [3].

All processes take place on a raster map, where each cell represents a functional classiﬁcation of a landscape denoting habitat quality. The cells of the model landscape represent about 4 km^2^, encompassing a boar group’s core home range [4]. Habitat quality of the grid cells is translated into breeding capacity, i.e. the number of female boars that are allowed to have offspring [5]. Thereby, local host density is regulated in the model (i.e. increasing numbers of fertile females can breed only until breeding capacity is approached).

The crucial model entity is the individual wild boar, characterised by age in weeks (where one week represents the approximate CSF incubation time [6,7]), resulting in age-classes: piglet (< 8 months ± 6 weeks), sub-adult (< 2 years ± 6 weeks) and adult. Each host individual has a location, which denotes its home range cell on the raster grid as well as its family group. Further, the individual wild boar comprises an epidemiological status (susceptible, transiently infected, lethally infected with individual infectious period or immune by surviving the infection or maternal antibodies). Sub-adult wild boars may disperse during the dispersal period (i.e. early summer) dependent on their demographic status (disperser or non-disperser).

## Process overview and scheduling

In the ﬁrst week of each year, females are assigned to breed according to the breeding capacity of their family group’s cell. Moreover, the model stochastically assigns natural survival ﬁgures to the running year. Thereby, ﬂuctuations in boar living conditions are represented, i.e. good and bad years.

Then, the model proceeds in weekly time steps. In each time step an ordered sequence of modules is executed: Infection and determination of the disease course, sub-adult dispersal, reproduction, death, and ageing. Figure 1 shows a ﬂow chart of the scheduling of submodels.

# Design concepts

Boar population dynamics emerge from individual behaviour, defined by age-dependent seasonal reproduction and mortality probabilities and age- and density-dependent dispersal behaviour, all including stochasticity. The epidemic course emerges from within and between group virus transmission, boar dispersal and individual stochastic disease courses and infectious periods for infected boars.

Demographic and behavioural parameters are represented by probability distributions to account for variations in the biological processes being modelled. Stochastic individual disease courses and infectious periods are modelled explicitly, as variation in disease outcome between individuals was identified as essential for virus endemicity without reservoirs [8].

# Details

## Initialization

The model landscape represents 200 km × 50 km of connected boar habitat without barriers. The 2500 grid cells are initialised randomly with uniformly distributed integer breeding capacity values *C_ij_* $\in$ {0, 1 …, 5}. The average breeding capacity is 2.5 females per cell, resulting in approximately 20 boars per cell or a host density of 5 boars per km^2^. Wild boar density reﬂects long-term average values of densely populated Central European habitats [9-12]. One boar group is allocated to each habitat cell. Group size was initialised as three times the breeding capacity of the habitat cell. Initial age distribution was obtained from the results of a 100-year model run, conducted by Kramer-Schadt et al. [8] (see Table 2).

## Input

The applied model setup does not include any external inputs or driving variables.

## Submodels

Submodels are described in the order of their execution. Parameters and their values are listed in Table 1.

### Virus release

The virus is released to the population by lethal infection of one randomly selected boar. The release is scheduled in a random week of the sixth year of the model run.

### Vaccination

Temporal schedule of vaccination campaigns over each year was equivalent for all simulations and followed existing protocols [12]: baiting campaigns are possible in fixed calendar weeks 20, 30 and 40 (end of May, early August, mid-October). Vaccination starts after virus release with the first possible campaign according to the temporal schedule and ends with the end of the simulation. Spatial application schemes were experimentally altered and are described in the section ‘Simulation experiments’. Bait uptake rates in terms of the proportion of hosts that receive at least one bait per campaign were assigned as $32~km$ *u^(a)^* = *u^(y)^* = 33% for adults and yearlings, and *u*$32~km$*^(p)^* = 5% for piglets (see Table 1). Pilet uptake rates were set low to represent difficulties in the vaccination of juveniles, particularly those younger than $32~km$4−5 months [12,13]. Bait uptake is evaluated independent of the animals’ epidemiologic or vaccination status. The resulting devolution of the proportion of immune hosts over time in a susceptible population is shown in Figure 2 and mimics respective field observations [14]. Immunity is life-long, and no booster effect is implemented in the model. Oral vaccination in wild boar is performed recently with C-strain filled baits. This modified live vaccine was repeatedly demonstrated to provide sterile immunity in all animals after eating a bait [3,15]. A marker vaccine which has yet been used experimentally for oral vaccination in the field has equivalent protective characteristics [16].

### Virus transmission

Transmission is modelled stochastically. The transmission parameter determines the weekly probability of being infected by an infectious group mate $P_{\inf}^{(i)}$. The probability of being infected by an infectious animal in one of the eight neighbouring groups, $P_{\inf}^{(e)}$, is ﬁxed as one tenth of $P_{\inf}^{(i)}$. For a susceptible animal the probability to become infected accumulates over all infectious animals within the group and in the neighbourhood according to

$$\Pi_{i}=1-(1-P_{\inf}^{(i)})^{\lambda_{i}}\cdot(1-P_{\inf}^{(e)})^{\Sigma_{j}\lambda_{j}}$$

where *λ_i_* is the number of infectious group mates and *λ_j_* is the numbers of infectious hosts in the *j^th^* neighbouring cell. The resulting probability value *Π_i_* provides the parameter of a binomial chance process to decide on becoming infected. Infection might be translocated within the host population during dispersal of sub-adult females.

The transmission parameter $P_{\inf}^{(i)}$ (and thereby $P_{\inf}^{(e)}$) was calibrated in order to reproduce the spreading velocity observed in France [17] with the simulated epidemic. The disease spread velocity of approx. 8 km per quarter was approached with the constant parameter value $P_{\inf}^{(i)}$ = 2.08 ∙ 10^-2^ within and, hence, $P_{\inf}^{(e)}$ = 2.08 ∙ 10^-3^ between groups.

### Disease course

CSF shows a variety of disease courses on the individual level [18,19]. Therefore, in our model the disease course is stochastically speciﬁed for each individual and determined by two parameters of probability distributions: individual case mortality *M* deﬁnes a binomial and the mean infectious period of all lethally infected hosts *μ* an exponential distribution.

The disease course sub model is described by two parameters: individual case mortality *M* and *μ*, the mean infectious period of lethally infected hosts. On infection the host is stochastically assigned either as lethally infected (with probability *M*) or as transiently infected (1 – *M*). *M* applies unchanged for yearlings *M^(y)^*, is decreased for adults to *M^(a)^* = *M*² and increased for piglets to *M^(a)^* = $\sqrt{M}$ to represent age-dependent disease outcomes [20].

Transiently infected wild boars ﬁrst pass through an infectious period of one week and subsequently become non-infectious but are protected against super-infection for the following three weeks. Afterwards antibodies are detectable and the animal is assigned to the immune state [6,7,12].

On infection lethally infected hosts receive their individual infectious period (*m* in weeks) drawn from the exponential distribution with the mean speciﬁed by parameter *μ*:

$$m=1+\left\lfloor-(\mu-0.5)\cdot\ln(U(0, 1))\right\rfloor$$

where $U(0, 1)$ is a uniformly distributed random number between 0 and 1. Lethally infected hosts remain infectious until death.

If the individual has maternal antibodies it is immune if its age *T_k_* is below the number of weeks of immunisation due to maternal antibodies *T_immune_*. Otherwise, mortality is decreased according

$$M_{MAB}=(1-M)+M\cdot\frac{1+T_{anti}-T_{k}}{T_{anti}-T_{immune}}$$

where *T_k_* is the individuals age in weeks, *T_anti_* the number of weeks maternal antibodies persist and *T_immune_* the number of weeks of immunisation due to maternal antibodies. A transient disease course is assigned stochastically by drawing a random number like described above.

### Herd splitting

Herd splitting is performed in specified weeks of the year only. The model collects subadults able to move for each of the *N* habitat cells, which are all female yearlings without offspring. Afterwards, all herds to split are extracted, matching the conditions of containing a number of females exceeding the cells breeding capacity and containing at least a specified number of subadults to move *N_disp_*.

Splittable herds are iterated randomly. For each of them, an empty habitat cell (breeding capacity above 0) within an Euclidean distance *D_disp_* is selected randomly, excluding the source cell. All migratable individuals from the considered herd of the source cell found a new herd on that cell.

### Reproduction

Females reproduce only once a year, depending on their age class (they have to be at least subadult). Individual females reproduce depending on the season with a peak in March and no reproduction in winter from October to December [21] (see ).

In the first week of the year, female individuals are checked whether they are able to breed. All females not exceeding their habitat cells breeding capacity, starting with the oldest individuals, are allowed to breed. The week of the year to breed is assigned according weekly reproduction probabilities, derived from monthly probabilities and the number of weeks in the month ().

Litter size is drawn from a precalculated truncated normal distribution () and reduced to a constant fraction for infected individuals. Litter size of transient shedders and lethally infected hosts is multiplied with the reduction factor *α_f_*.

Depending on the disease state of the breeding individual, its piglet’s disease states are adjusted. Susceptible individuals and transient non-shedders produce susceptible offspring, immune individuals (no matter if due to preceding infection or vaccination) produce immune offspring with maternal antibodies. Transient shedders and lethally infected individuals yield offspring, each one lethally infected with a given probability of prenatal infection *P_PI_*.

### Mortality

Iterating over the entire population, each individual either stochastically dies due to age class dependent mortality rates by drawing a random number, due to reaching a certain maximum age, or due to a lethal infection after a certain infection time span *m* (see section “Infection”).

Stochastic baseline mortality is age-dependent and adjusted to annual survival estimates found in the literature. These survival estimates together with the reported variability (see Table 1) determine the Gaussian distributions we draw from the random survival in the model on a yearly basis (*SP_Year_*). The stochastic effect resembles “good” or “bad” years for boars, i.e. environmental noise. In the application the Gaussian distributions are cut symmetrically around the mean. Per time step we apply the adjusted age-dependent mortality (*PM_Week_*) to the individual:

$PM_{Week}=1-(SP_{Year})^{1/52}$.

### Ageing

The ageing process iterates over all individuals. For each individual *k*, age *T_k_* is incremented one week and disease state transitions are performed. Transient shedders are converted to transient non-shedders after a certain infectious period *T_trans_*, losing their maternal antibodies if present. Transient non-shedder convert to immune after a certain latency period *T_latent_*.

An individual *k* protected by maternal antibodies turns susceptible if reaching an age *T_k_* of the protection time *T_immune_*. Maternal antibodies themselves vanish on reaching an age of maternal antibodies persistence *T_anti_*.

After disease state transition the age of the infection is incremented one week if the individual is not susceptible.

# Parameters, simulation experiments, analysis

## Independent variables

There are two independent variables in our analysis. Lethality of infection is defined by individual case mortality parameter $32~km$*M*, and life-expectancy of lethally infected hosts is specified by the parameter *$32~km$μ*. All simulation experiments were performed for $32~km$ and to cover a wide range, no matter whether the extremes are biologically meaningful$32~km$. The range covers lethality from 0 – 100 % and mean infectious periods after lethal infection as long as 10 weeks. With that, we recognize the reported heterogeneity in these two parameters as measured in the field [19,22,23].

The individual disease courses were scaled up to the whole population to measure the effective mean infectious period *T*_inf_$32~km$.

## Simulation experiments

Four spatial vaccination schemes (Figure 3), and a non-vaccination reference were applied. The vaccination schemes were motivated by different level of accuracy in following the actually infected area:

0. “No vaccination”: reference with no baiting at all.

1. “Complete vaccination”: baiting is applied to the entire landscape (Figure 3a).

2. “Actually infected area vaccination”: baiting is applied on all habitat cells that are infected in the week of the recent campaign (Figure 3b).

3. “Ever infected area vaccination”: baiting is applied on all habitat cells that have been infected in the given model run (or recent outbreak, Figure 3c). The strategy is comparable to recent baiting strategies of successive vaccination zone extending with disease spread.

4. “Buffered vaccination”: baiting is applied on all habitat cells that are infected in the week of the campaign and a buffer of 32 km around them (Figure 3d). The buffer radius of 32 km is motivated by the saturation of the proportion of immune hosts after three campaigns, i.e. one year (Figure 2) and the spreading velocity of the epidemic wave of 8 km per quarter, i.e. 32 km per year.

For each vaccination scheme and each *M* × *µ*$32~km$ combination $32~km$120 model runs were conducted to achieve a minimum precision of $32~km$± 9% with $32~km$95% confidence for proportions, resulting in $32~km$13 200 runs per scheme.

Simulations were performed for 20 years or until host or virus became extinct. In detail, the virus was released into the boar population in a random week of the sixth year by infection of one randomly selected boar individual and then simulations continued up to maximum further 14 years.

## Dependent variables

The simulation output focused on two dependent variables: (1) the extent of the outbreak as measured by the maximum distance from the release point, and (2) the risk of endemicity as measured by the probability of virus circulation after 10 years.

Maximum virus distance from the release point *D*_max_$32~km$ was recorded as a measure of disease spread. The average maximum distance from a randomly selected release point was about 155 km and is defined by the most distant corner of the landscape. In detail, for a landscape of 200 km × 50 km$32~km$, average maximum edge distances are 150 km$32~km$ and 37.5 km$32~km$, resulting in an average possible distance of $32~km$ if spread always covers the full landscape.

Virus persistence was measured in weeks since virus release. Individual runs were labelled endemic if the virus is present after 10 years and non-endemic for earlier virus fade-out. The proportion is then described by the dependent variable *P_end_*$32~km$ measuring probability of endemicity from $32~km$120 repetitions of a simulation scenario.

## Analysis

Data was analysed by applying contour plots of response variables using *M*$32~km$ and *µ*$32~km$ as X resp. Y axis. To identify the parameter scopes of the different effects of the schemes tested, differences to the reference scenario were calculated.

Analysis was performed using GNU R 2.9.2 (R Core Development Team); plots were created with SigmaPlot$32~km$® 10.0 (Systat Software Inc.).

**References**

1. Grimm V, Berger U, Bastiansen F, Eliassen S, Ginot V, Giske J, Goss-Custard J, Grand T, Heinz S, Huse G, Huth A, Jepsen JU, Jørgensen C, Mooij WM, Müller B, Pe'er G, Piou C, Railsback SF, Robbins AM, Robbins MM, Rossmanith E, Rüger N, Strand E, Souissi S, Stillman RA, Vabø R, Visser U, DeAngelis DL: **A standard protocol for describing individual-based and agent-based models.** *Ecol Model* 2006, **192**:115-126.

2. Grimm V, Berger U, DeAngelis DL, Polhill JG, Giske J, Railsback SF: **The ODD protocol: A review and first update.** *Ecol Model* 2010, **221**:2760-2768.

3. Laddomada A: **Incidence and control of CSF in wild boar in Europe.** *Vet Microbiol* 2000, **73**:121-130.

4. Leaper R, Massei G, Gorman ML, Aspinall R: **The feasibility of reintroducing Wild Boar (*Sus scrofa*) to Scotland.** *Mammal Rev* 1999, **29**:239-258.

5. Jedrzejewska B, Jedrzejewski W, Bunevich AN, Milkowski L, Krasinski ZA: **Factors shaping population densities and increase rates of ungulates in Bialowieza Primeval Forest (Poland and Belarus) in the 19th and 20th centuries.** *Acta Theriol* 1997, **42**:399-451.

6. Artois M, Depner KR, Guberti V, Hars J, Rossi S, Rutili D: **Classical swine fever (hog cholera) in wild boar in Europe.** *Rev Sci Tech* 2002, **21**:287-303.

7. Moenning V, Floegel-Niesmann G, Greiser-Wilke I: **Clinical signs and epidemiology of classical swine fever: A review of a new Knowledge.** *Vet J* 2003, **165**:11-20.

8. Kramer-Schadt S, Fernández N, Eisinger D, Grimm V, Thulke H-H: **Individual variations in infectiousness explain long-term disease persistence in wildlife populations.** *Oikos* 2009, **118**:199-208.

9. Howells O, Edwards-Jones G: **A feasibility study of reintroducing wild boar *Sus scrofa* to Scotland: are existing woodlands large enough to support minimum viable populations.** *Biol Conserv* 1997, **81**:77-89.

10. Sodeikat G, Pohlmeyer K: **Escape movements of family groups of wild boar *Sus scrofa* influenced by drive hunts in Lower Saxony, Germany.** *Wildl Biol* 2003, **9**(Suppl. 1):43-49.

11. Melis C, Szafranska PA, Jedrzejewska B, Barton K: **Biogeographical variation in the population density of wild boar (*Sus scrofa*) in western Eurasia.** *J Biogeogr* 2006, **33**:803-811.

12. EFSA: **Control and eradication of Classic Swine Fever in wild boar - Scientific Opinion.** *EFSA J* 2009, **932**:1-18.

13. Kaden V, Lange E, Fischer U, Strebelow G: **Oral immunisation of wild boar against classical swine fever: evaluation of the first field study in Germany.** *Vet Microbiol* 2000, **73**:239-252.

14. von Rüden SM, Staubach C, Kaden V, Hess RG, Blicke J, Kühne S, Sonnenburg J, Fröhlich A, Teuffert J, Moenning V: **Retrospective analysis of the oral immunisation of wild boar populations against classical swine fever virus (CSFV) in region Eifel of Rhineland-Palatinate.** *Vet Microbiol* 2008, **132**:29-38.

15. Kaden V, Lange B: **Oral immunisation against classical swine fever (CSF): onset and duration of immunity.** *Vet Microbiol* 2001, **82**:301-310.

16. **CSFV_goDIVA** [http://www.csfvaccine.org/]

17. Rossi S, Pol F, Forot B, Masse-provin N, Rigaux S, Bronner A, LePotier M-F: **Preventive vaccination contributes to control classical swine fever in wild boar (*Sus scrofa sp.*).** *Vet Microbiol* 2010, **142**:99-107.

18. Liess B: **Pathogenesis and epidemiology of hog cholera.** *Ann Rech Vet* 1987, **18**:139-145.

19. Depner KR, Hinrichs U, Bickhardt K, Greiser-Wilke I, Pohlenz J, Moenning V, Liess B: **Influence of breed-related factors on the course of classical swine fever virus infection.** *Vet Rec* 1997, **140**:506-507.

20. Dahle J, Liess B: **A review of classical swine fever infections in pigs: epizootiology, clinical disease, and pathology.** *Comp Immunol Microbiol Infect Dis* 1992, **15**:203-211.

21. Boitani L, Trapanese P, Mattei L, Nonis D: **Demography of a wild boar (*Sus scrofa* L.) population in Tuscany.** *Gibier Faune Sauvage* 1995, **12**:109-132.

22. Kaden V, Lange E, Polster U, Klopfleisch R, Teifke JP: **Studies on the virulence of two field isolates of the classical Swine Fever virus genotype 2.3 *Rostock* in wild boars of different age groups.** *J Vet Med B Infect Dis Vet Public Health* 2004, **51**:202-208.

23. Kaden V, Ziegler U, Lange E, Dedek J: **Classical swine fever virus: clinical, virological, serological and hematological findings after infection of domestic pigs and wild boars with the field isolate "Spante" originating from wild boar.** *Berl Münch Tierärztl Wochenschr* 2000, **113**:412-416.

24. Jezierski W: **Longevity and mortality rate in a population of wild boar.** *Acta Theriol* 1977, **22**:337-348.

25. Depner K, Müller T, Lange E, Staubach C, Teuffert J: **Transient classical swine fever virus infection in wild boar piglets partially protected by maternal antibodies.** *Dtsch Tierarztl Wochenschr* 2000, **107**:66-68.

26. Focardi S, Toso S, Pecchioli E: **The population modelling of fallow deer and wild boar in a Mediterranean ecosystem.** *For Ecol Manage* 1996, **88**:7-14.

27. Gaillard J-M, Vassant J, Klein F: **Some characteristics of the population dynamics of wild boar (*Sus scrofa scrofa*) in a hunted environment.** *Gibier Faune Sauvage* 1987, **4**:31-47.

28. Bieber C, Ruf T: **Population dynamics in wild boar *Sus scrofa*: ecology, elasticity of growth rate and implications for the management of pulsed resource consumers.** *J Appl Ecol* 2005, **42**:1203-1213.

Table 1: Model parameters

| **Symbol** | **Description** | **Value** | **Source / details** |
| --- | --- | --- | --- |
| $\alpha_{f}$ | Fertility reduction if ill | 0.625 |  |
| $P_{PI}$ | Probability of prenatal infection | 0.5 |  |
| $T_{\max}$ | Maximum age of a boar | 11 years (572 weeks) | [24] |
| $T_{anti}$ | Maximum persistence of maternal antibodies | 15 weeks | [25] |
| $T_{immune}$ | Maximum duration of immunity by maternal antibodies | 12 weeks | [25] |
| $T_{trans}$ | Infectious period of transiently infected hosts | 1 week | [6,7] |
| $T_{AB}$ | Period of transiently infected between infectious period and detectable antibodies | 3 weeks | [12] |
| $P_{\inf}^{(i)}$ $P_{\inf}^{(e)}$ | Infection probability within / between herds |  | Reversely fitted to the estimated disease spread velocity of approx. 8 km per quarter [17] |
| $M$ | Case mortality (subadults, see section “Disease course”) | (0.0, 0.1, …, 1.0 ) |  |
| $\mu$ | Expectation value of the exponential distribution of life expectancy of lethally infected hosts | (1, 2, …, 10) weeks |  |
| $D_{disp}$ | Maximum dispersal distance for subadult females | 6 km | [10] |
| $N_{disp}$ | Minimum number of subadult females for dispersal | 2 |  |
| $SP_{mean}^{(a)}/SP_{\min}^{(a)}$ | Mean / minimum annual survival rate adults | 0.65 / 0.4 | [26] |
| $SP_{mean}^{(y)}/SP_{\min}^{(y)}$ | Mean / minimum annual survival rate yearlings | 0.65 / 0.4 | [27] |
| $SP_{mean}^{(p)}/SP_{\min}^{(p)}$ | Mean / minimum annual survival rate piglets | 0.5 / 0.1 | [26] |
| $u^{(a)}$ $u^{(y)}$ $u^{(p)}$ | Bait uptake rates adults / yearlings / piglets | 0.33  0.33  0.05 | Low piglet uptake rates: difficulties in vaccination of juveniles, younger than 4 - 5 months [12,13]. |

Table 2: Initial age distribution [8].

| Upper age bound (years) | 1 | 2 | 3 | 4 | 5 | 6 | 7 | 8 | 9 | 10 | 11 |
| --- | --- | --- | --- | --- | --- | --- | --- | --- | --- | --- | --- |
| Proportion | 0.38 | 0.24 | 0.15 | 0.09 | 0.06 | 0.03 | 0.02 | 0.01 | 0.01 | 0.01 | 0.00 |

Table 3: Monthly reproduction probabilities [21].

| Month | Jan | Feb | Mar | Apr | May | Jun | Jul | Aug | Sep | Oct | Nov | Dec |
| --- | --- | --- | --- | --- | --- | --- | --- | --- | --- | --- | --- | --- |
| Num. weeks | 4 | 4 | 5 | 4 | 4 | 5 | 4 | 4 | 5 | 5 | 4 | 4 |
| Reprod. prob. | 0.0 | 0.10 | 0.23 | 0.34 | 0.07 | 0.08 | 0.06 | 0.03 | 0.03 | 0.00 | 0.00 | 0.00 |

Table 4: Breed count distribution [28].

| Num. offspring | 0 | 1 | 2 | 3 | 4 | 5 |
| --- | --- | --- | --- | --- | --- | --- |
| Probability | 0.01306 | 0.06915 | 0.016290 | 0.24994 | 0.24994 | 0.016290 |
| Num. offspring | 6 | 7 | 8 | 9 | 10 |  |
| Probability | 0.06915 | 0.01910 | 0.00343 | 0.00040 | 0.00002 |  |


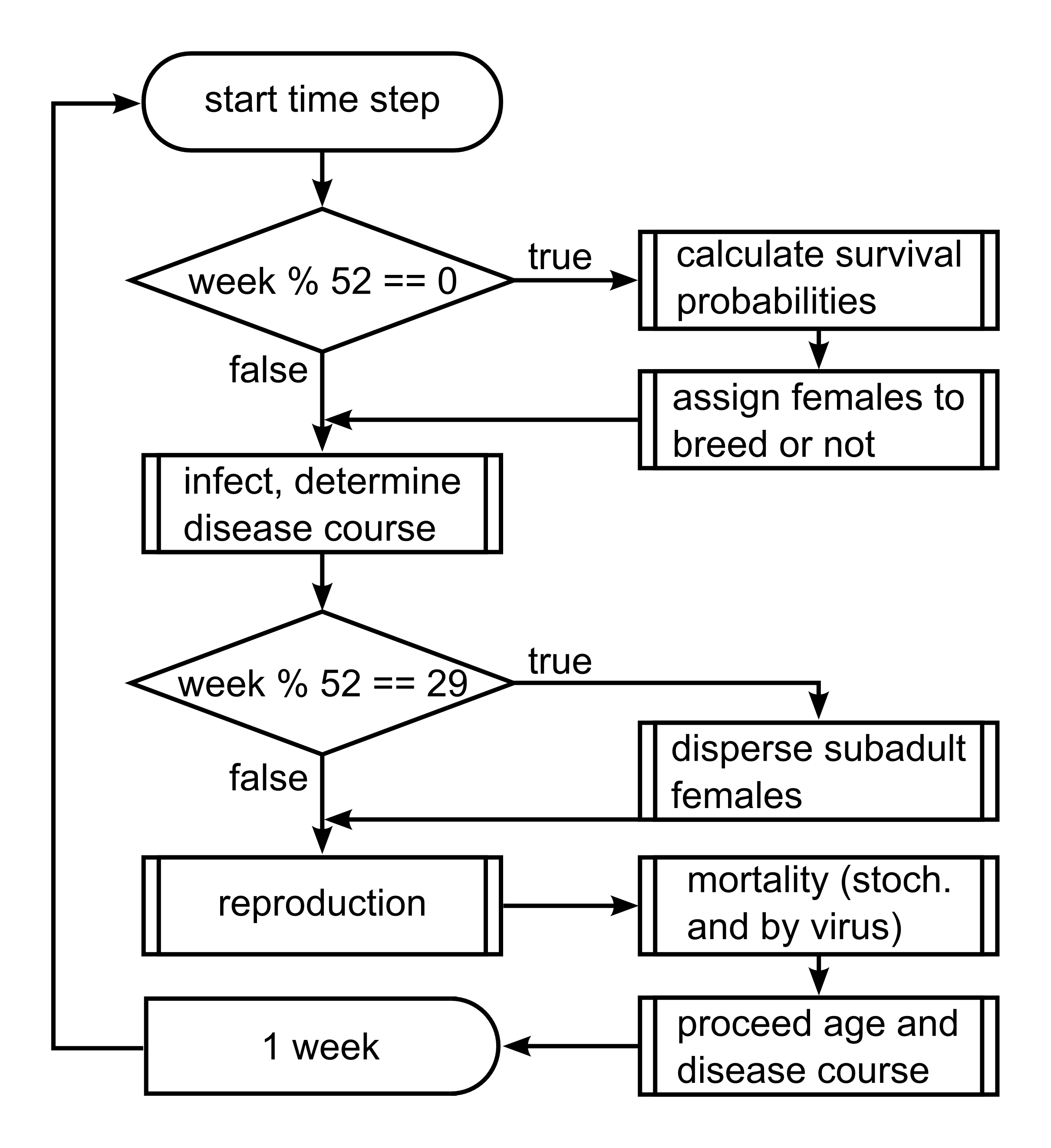


Figure 1: Flow chart of the scheduling of sub-models.


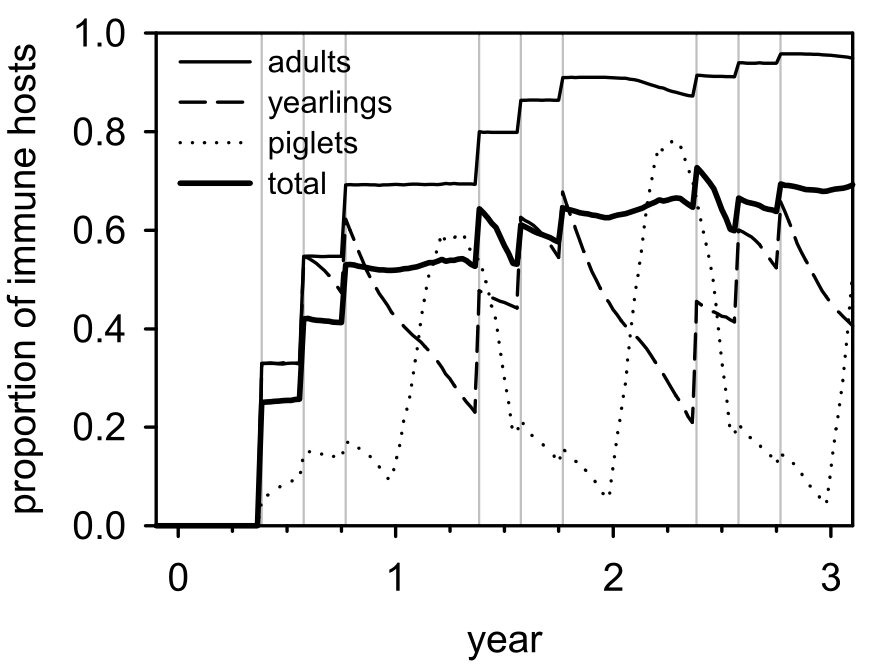


Figure 2: Time series of proportion of immune hosts due to oral mass vaccination (total and for age classes). Vertical grey lines denote dates of baiting campaigns.


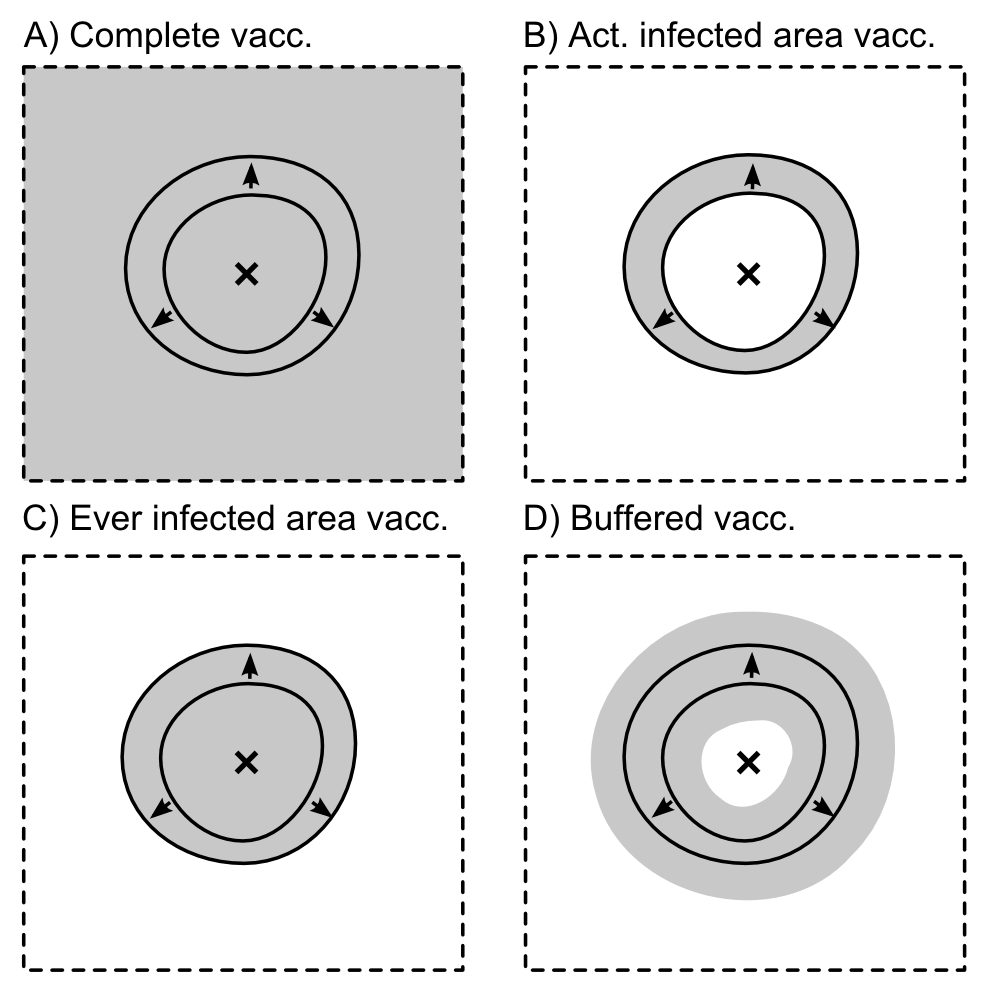


Figure 3: Schematic diagram of spatial vaccination strategies with infected area (solid outlines), baiting zone (grey fill), virus release point (cross) and disease spread direction (arrows). The circular pattern of infected areas is idealized from more distorted model outcomes.
