# Supplementary material for: Efficiency of spatio-temporal vaccination regimes in wildlife populations under different viral constraints
Source: Vet Res. 2012 Apr 24;43(1):37. doi: 10.1186/1297-9716-43-37 (PMC3384476; doi:10.1186/1297-9716-43-37)
Supplement: Additional file 2 — Sensitivity analysis. [file 1297-9716-43-37-S2.DOC]

**Supplementary material**

**Appendix 2: Sensitivity analysis**

# Introduction

Besides virulence parameters *M* and *μ*, which were extensively investigated in the main article, infection parameters ($P_{\inf}^{(i)}$,$P_{\inf}^{(e)}$) were the most uncertain model parameters in the study. Therefore, a sensitivity analysis regarding infection probabilities and the ratio between infection probabilities between and within herds was conducted.

# Materials and Methods

## Parameters, simulation experiments, analysis

### Simulation experiments

Using submodels and parameters described in the main article, a sensitivity analysis on the infection parameters within and between herds ($P_{\inf}^{(i)}$,$P_{\inf}^{(e)}$) was conducted. For this purpose, $P_{\inf}^{(i)}$ was varied, taking values (0.0104, 0.0208, 0.104) while keeping the ratio $P_{\inf}^{(e)}$ / $P_{\inf}^{(i)}$ constant (1/10). For the default infection parameter within herds ($P_{\inf}^{(i)}=0.0208$), the ratio $P_{\inf}^{(e)}$ / $P_{\inf}^{(i)}$ was varied taking values (1/100, 1/10, 1/1). For each of the five resulting combinations of $P_{\inf}^{(e)}$ and $P_{\inf}^{(i)}$ (see scheme in Figure 1 bottom left), vaccination strategies as explained in the article were simulated.

For each vaccination scheme, each infection parameter set and each *M × µ* combination 40 model runs were conducted, resulting in 110 000 runs.

The buffer radius for the buffered vaccination scheme was adapted for each combination of $P_{\inf}^{(e)}$ and $P_{\inf}^{(i)}$ to fit the spreading distance of the virus within one year under the given infection parameter set.

Simulations were performed for 20 years or until host or virus became extinct. The virus was released into the boar population in a random week of the sixth year by infection of one randomly selected boar individual.

### Analysis

Data was analysed by applying contour plots of response variables using *M* and *µ* as X resp. Y axis. To identify the parameter scopes of the different effects of the schemes tested, differences to the reference scenario were calculated.

Analysis was performed using GNU R 2.9.2 (R Core Development Team); plots were created with SigmaPlot® 10.0 (Systat Software Inc.).

# Results

Increased infection probability within as well as between herds resulted in spread of the virus over the whole landscape for the entire explored parameter space (top and right in Figure 1). Decreased infection probability within as well as between herds resulted in a limitation of considerable virus spread to increased effective mean infectious periods (left and bottom in Figure 1). Increased infection probability within as well as between herds resulted in an extended scope of virus persistence, while decreased infection probabilities limited the scope to increased effective mean infectious periods (Figure 2). The pattern of virus persistence attributed to sufficiently long infectious periods and limitation of persistence by high case mortalities is applicable for all tested infection probabilities. Only for low infection probability between herds, reduction of persistence by high case mortalities is not visible. Here, the time horizon of 10 years was too short to measure probability of endemicity, since the spread speed of the epidemic wave was as short as 12 km per year.

Maximum virus spread and probability of virus endemicity under baiting of the entire landscape are shown in Figure 3 and Figure 4, respectively. With increased infection probability within as well as between herds, baiting was not capable to limit virus spread. For all other sets of infection parameters, vaccination limited virus spread to increased effective mean infectious periods. With increased infection probability within as well as between herds, vaccination could not limit virus persistence, but increased the persistence probability for high case mortalities. For all other sets of infection parameters, vaccination decreased probability of virus persistence for short effective mean infectious periods, but increased probability of virus persistence at high case mortalities.

The effect of buffered baiting equals the effect of complete baiting for maximum virus spread (compare Figure 5 and Figure 6) as well as probability of virus persistence (compare Figure 7 and Figure 8) under all tested combinations of infection parameters.

Baiting of areas infected during the outbreak as well as baiting of the actually infected area had a negligible effect on virus spread (Figure 9 and Figure 10). Baiting of areas affected during the outbreak had a weak effect on the probability of virus endemicity (Figure 11). Baiting of the actually infected area had a negligible effect on the probability of virus endemicity (Figure 12).

# Discussion

The principal mechanisms leading to successful virus spread and to endemicity were independent of the parameterisation of the transmission probabilities: Successful spread requires sufficiently long effective mean infectious periods *T*_inf_. Transition to endemicity requires sufficiently long effective mean infectious periods, i.e. successful spread, and is restricted to limited lethality. For details see section ‘Discussion’ in the main article.

Whenever baiting had a considerable impact on virus spread and probability of virus persistence, buffered vaccination resembled vaccination of the entire landscape.

Baiting of areas infected during the outbreak as well as baiting of the actually infected area have marginal or negligible effects on virus spread and probability of virus persistence for all tested combinations of infection probabilities.

In conclusion, the sensitivity analysis regarding transmission parameters revealed unchanged model outcome compared to the default set of infection parameters. The main findings regarding the performance of different spatial baiting schemes, therefore, are independent of the parameterisation of the transmission probabilities.


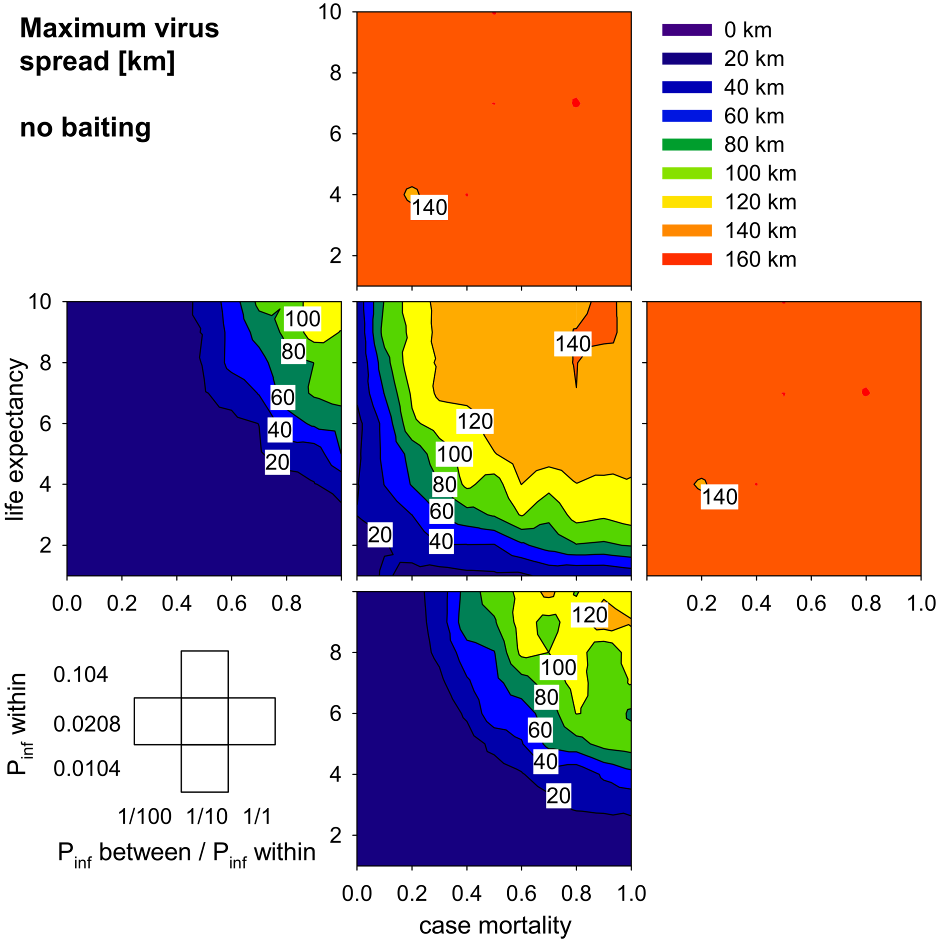


Figure 1: Median of the maximum achieved distance of the virus from the release point *D*_max_ without vaccination.


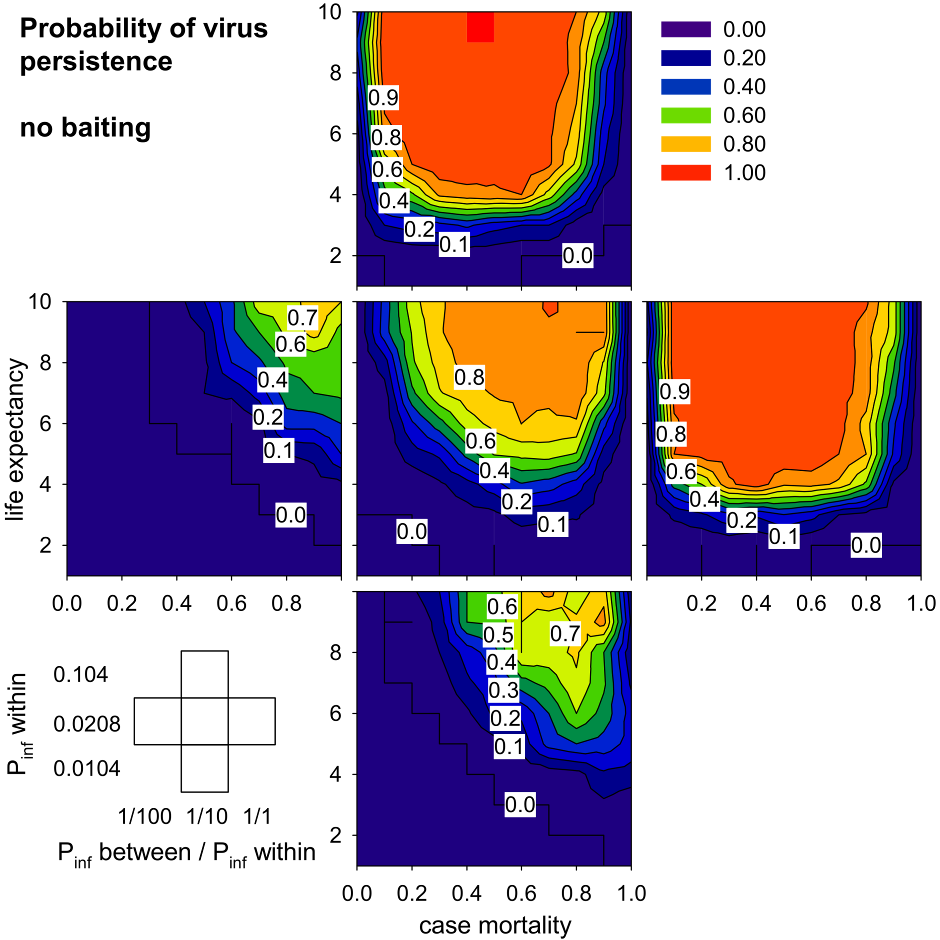


Figure 2: Probability of virus endemicity *P*_end_ without vaccination.


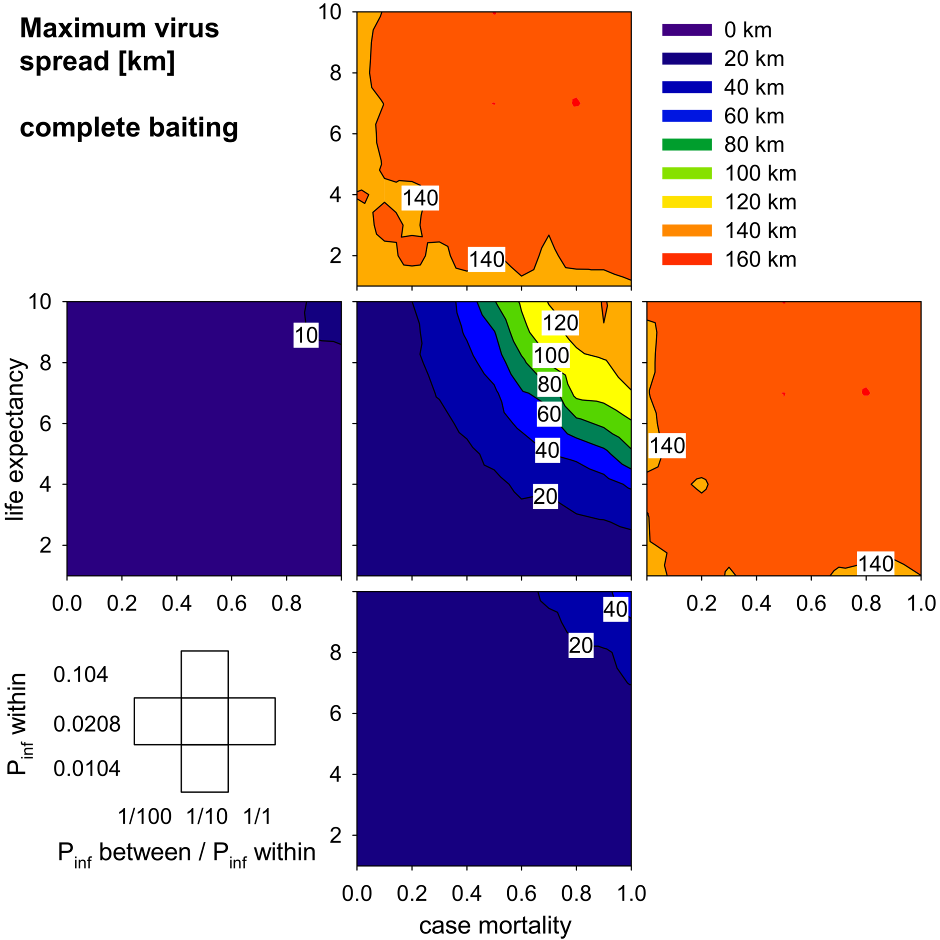


Figure 3: Median of the maximum achieved distance of the virus from the release point *D*_max_ with complete baiting.


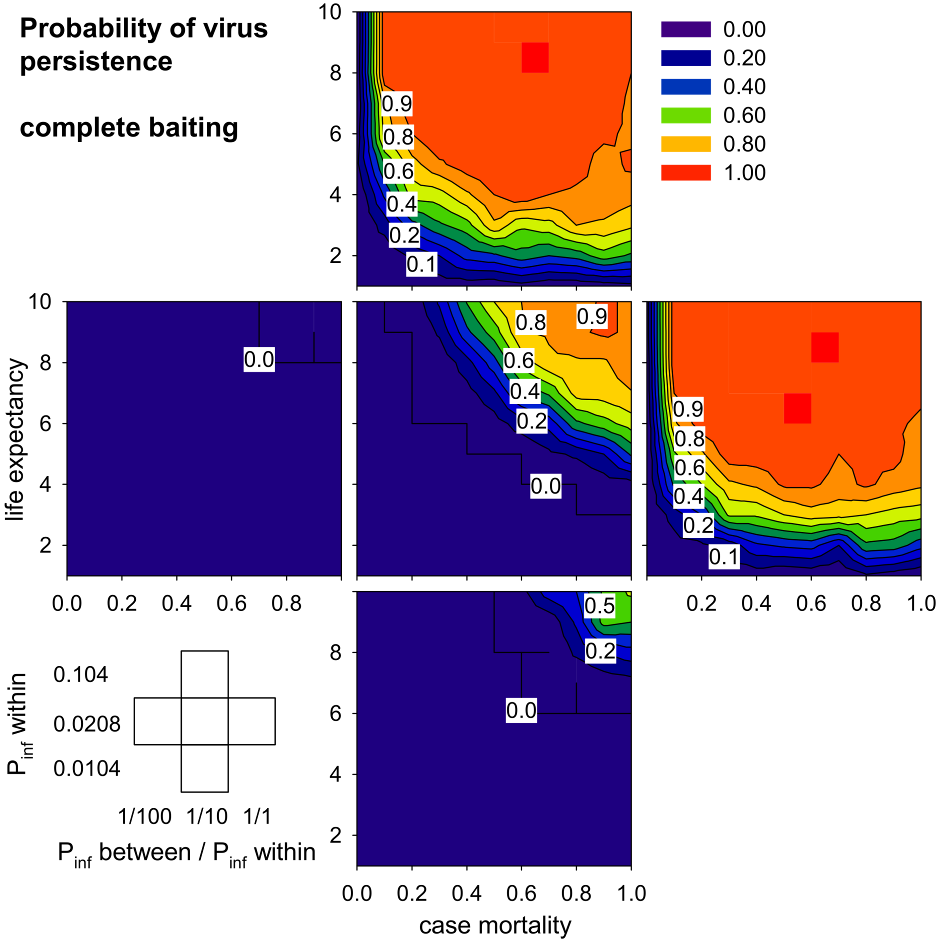


Figure 4: Probability of virus endemicity *P*_end_ with complete baiting.


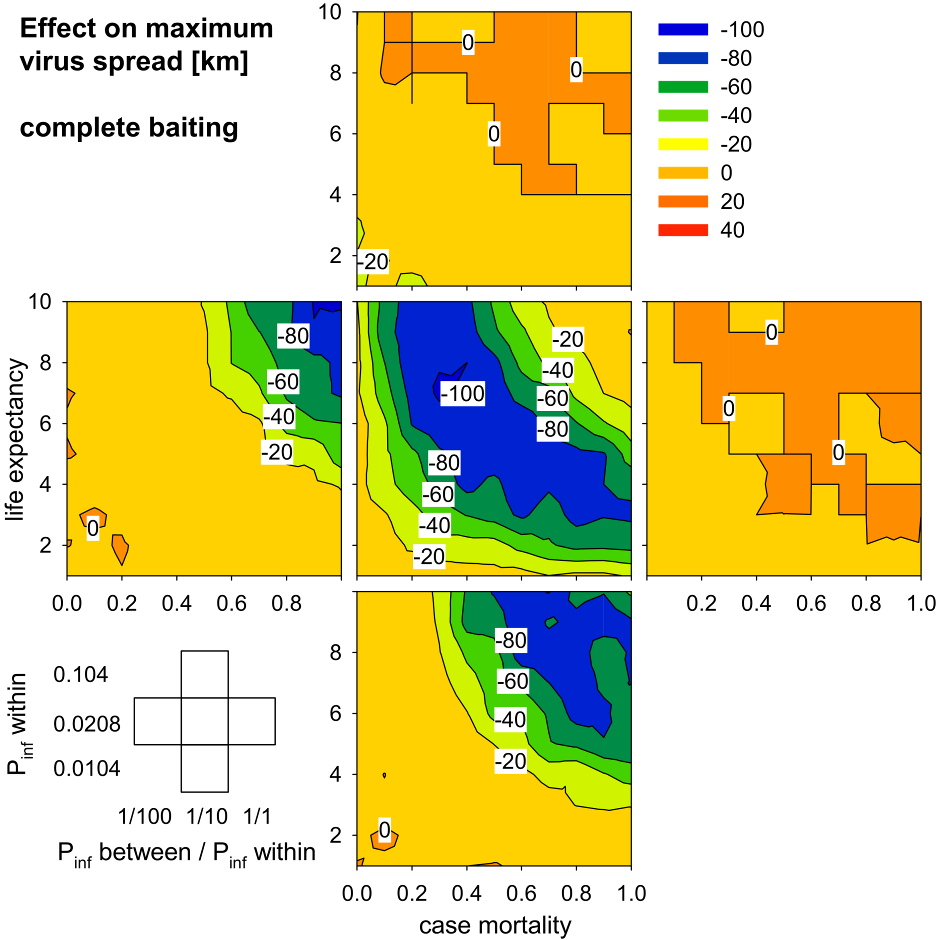


Figure 5: Effect of complete baiting on maximum achieved distance of the virus from the release point $\Delta D_{\max}$ (pointwise difference between Figure 1 and Figure 3).


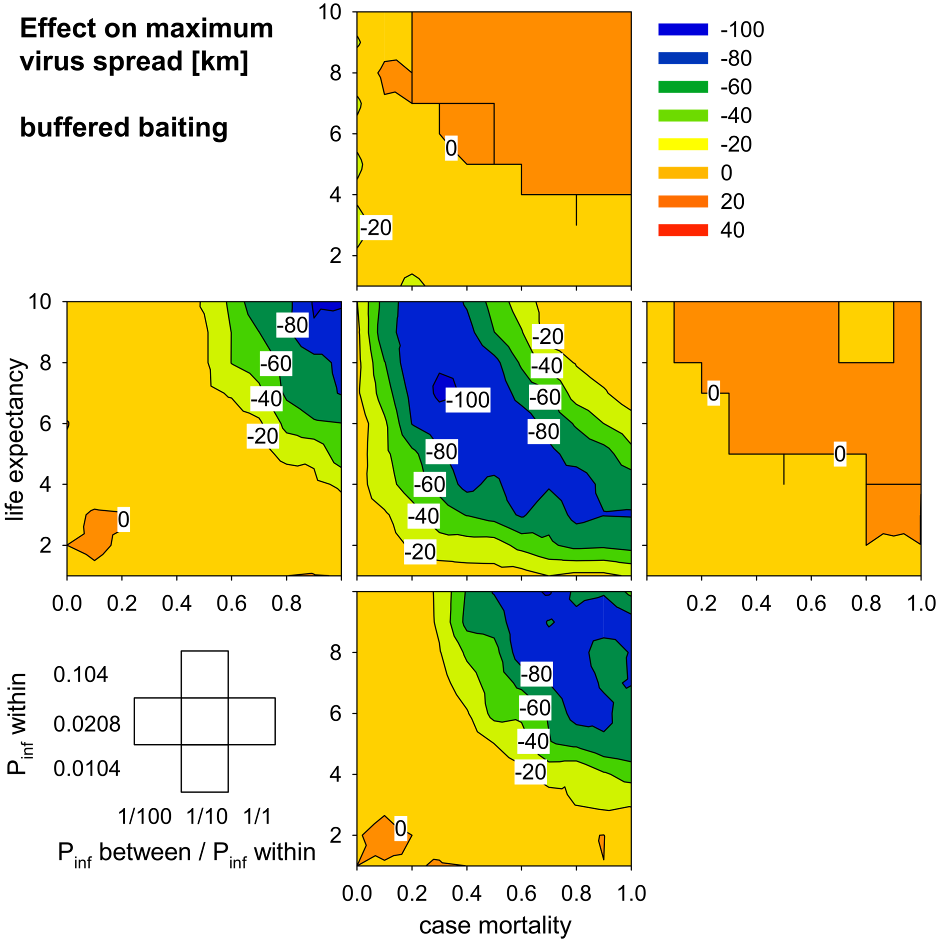


Figure 6: Effect of buffered baiting on maximum achieved distance of the virus from the release point $\Delta D_{\max}$.


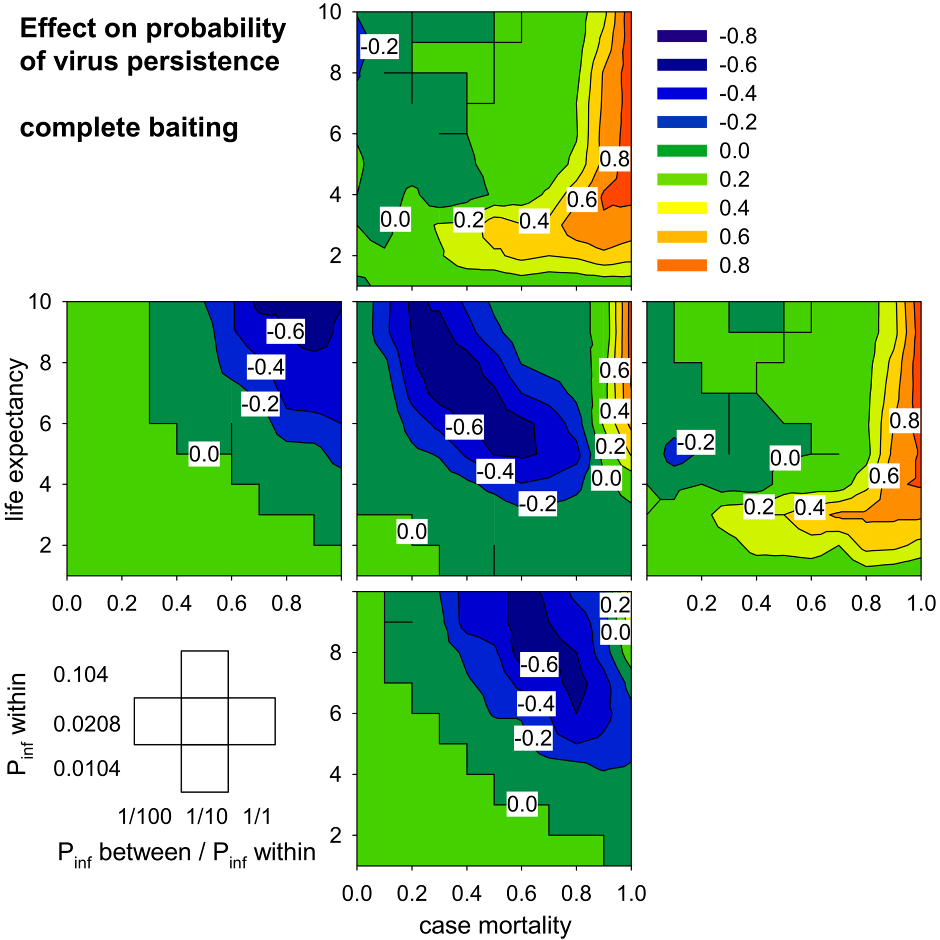


Figure 7: Effect of complete baiting on probability of virus endemicity $\Delta P_{\text{end}}$ (pointwise difference between Figure 2 and Figure 4).


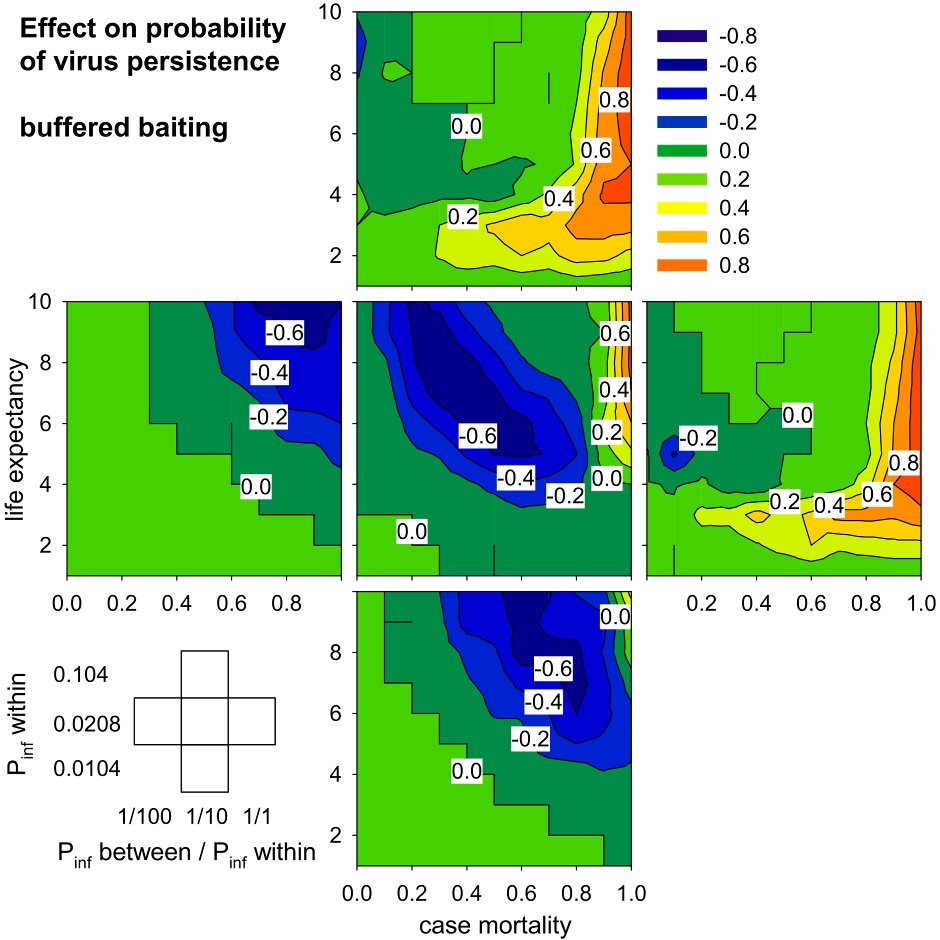


Figure 8: Effect of buffered baiting on probability of virus endemicity $\Delta P_{\text{end}}$.


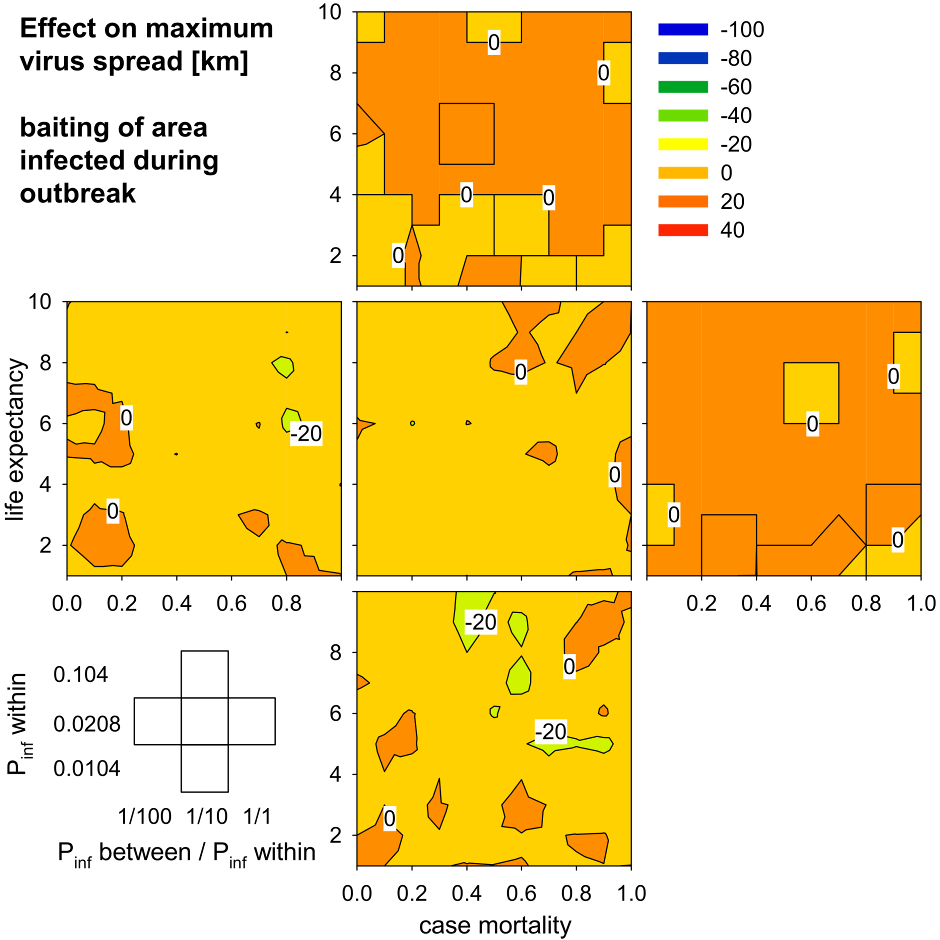


Figure 9: Effect of baiting of area infected during outbreak on maximum achieved distance of the virus from the release point $\Delta D_{\max}$.


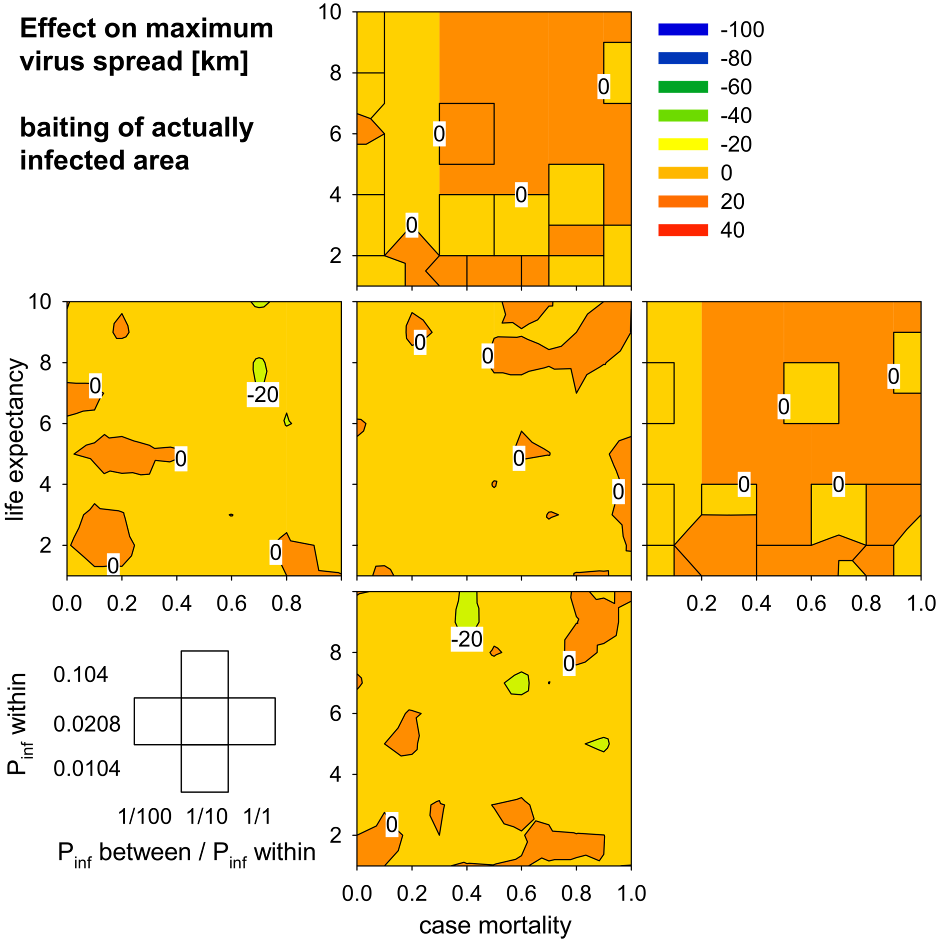


Figure 10: Effect of baiting of actually infected area on maximum achieved distance of the virus from the release point $\Delta D_{\max}$.


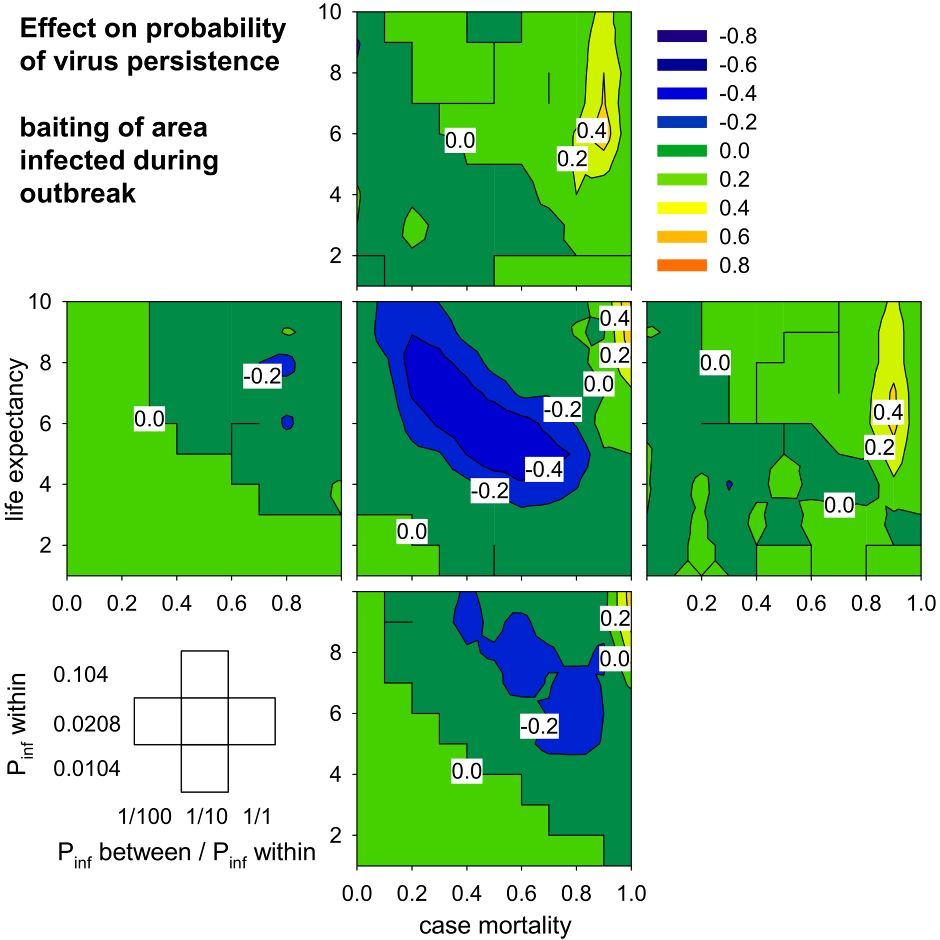


Figure 11: Effect of baiting of area infected during outbreak on probability of virus endemicity $\Delta P_{\text{end}}$.


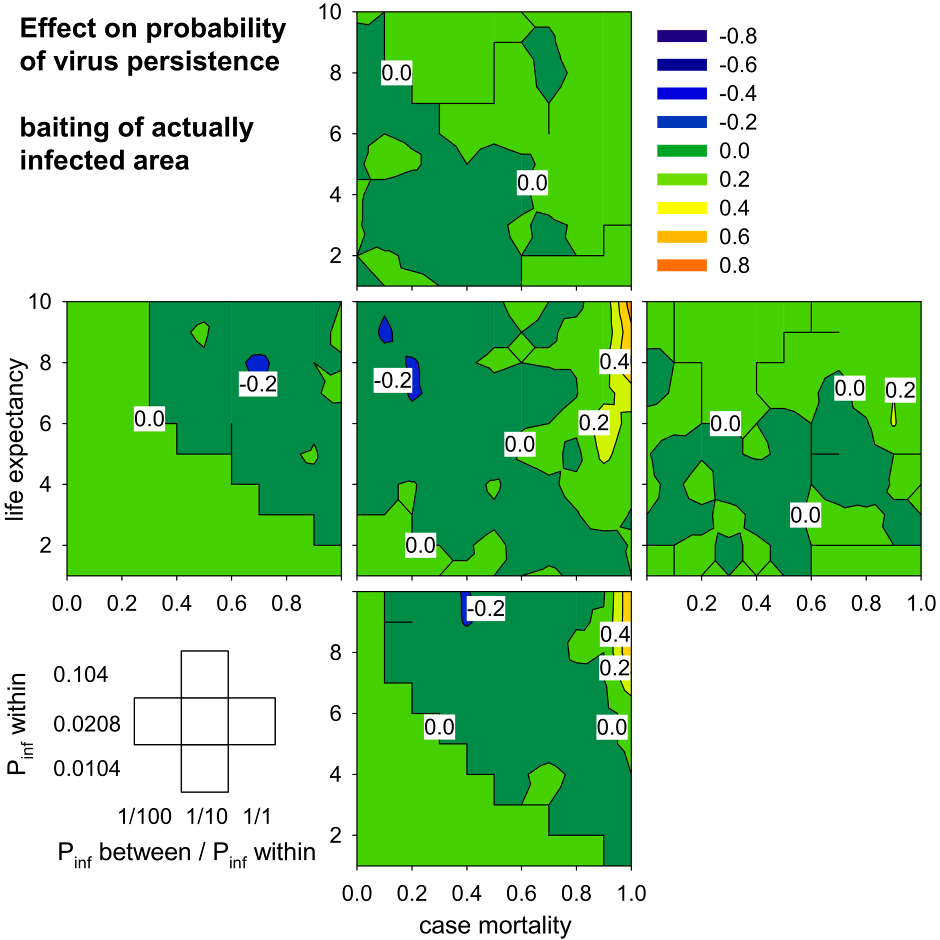


Figure 12: Effect of baiting of actually infected area on probability of virus endemicity $\Delta P_{\text{end}}$.
